# Biomarkers of environmental enteric dysfunction and adverse birth outcomes: An observational study among pregnant women living with HIV in Tanzania

**DOI:** 10.1016/j.ebiom.2022.104257

**Published:** 2022-09-18

**Authors:** Miles A. Kirby, Jacqueline M. Lauer, Alfa Muhihi, Nzovu Ulenga, Said Aboud, Enju Liu, Robert K.M. Choy, Michael B. Arndt, Jianqun Kou, Andrew Gewirtz, Wafaie W. Fawzi, Christopher P. Duggan, Karim P. Manji, Christopher R. Sudfeld

**Affiliations:** a Department of Global Health and Population, Harvard T.H. Chan School of Public Health, Boston, MA, United States; b Department of Health Sciences, Sargent College, Boston University, Boston, MA, United States; c Management and Development for Health, Dar es Salaam, Tanzania; d Department of Microbiology and Immunology, Muhimbili University of Health and Allied Sciences, Dar es Salaam, Tanzania; e Division of Gastroenterology, Hepatology and Nutrition, Boston Children's Hospital, Boston, MA, United States; f PATH, Center for Vaccine Innovation and Access, Seattle, WA, United States; g Institute for Health Metrics and Evaluation, University of Washington, Seattle, WA, United States; h Institute for Biomedical Sciences, Georgia State University, Atlanta, GA, United States; i Department of Epidemiology, Harvard T.H. Chan School of Public Health, Boston, MA, United States; j Department of Nutrition, Harvard T.H. Chan School of Public Health, Boston, MA, United States; k Department of Pediatrics and Child Health, Muhimbili University of Health and Allied Sciences, Dar es Salaam, Tanzania

**Keywords:** Biomarkers, Birth outcomes, Prenatal nutrition, Environmental enteric dysfunction, Inflammation, Growth hormone resistance

## Abstract

**Background:**

Environmental enteric dysfunction (EED) may contribute to adverse birth outcomes in low-resource settings. We examined the associations of EED biomarkers with birth outcomes in pregnant women living with human immunodeficiency virus in Dar es Salaam, Tanzania.

**Methods:**

We performed a cohort study of 706 HIV-infected pregnant women. Maternal serum samples collected at 32 weeks gestation were analyzed for markers of EED (anti-flagellin and anti-LPS immunoglobulins, intestinal fatty acid-binding protein [I-FABP] and soluble CD14), systemic inflammation (C-reactive protein and α1-acid glycoprotein [AGP]), and growth hormone resistance (insulin-like growth factor 1 [IGF-1] and fibroblast growth factor 21 [FGF21]. Associations of biomarkers categorized into quartiles with birth outcomes (birthweight, gestational duration, birthweight-for-gestational age, and stillbirth) were assessed using linear and log-binomial regression models adjusted for multiple sociodemographic and clinical variables.

**Findings:**

Maternal EED biomarkers were not significantly associated with birthweight, gestation duration, or birthweight-for-gestational age. However, higher quintiles of I-FABP concentrations were associated with greater risk of stillbirth (p_trend_=0·02). Higher AGP was associated with lower birthweight and was associated with increased risk of small-for-gestational age births. Higher IGF-1 was associated with higher birthweight and birthweight-for-gestational age while higher FGF21 was associated with shorter gestation and higher risk of preterm birth.

**Interpretation:**

Maternal biomarkers of EED, systemic inflammation, and growth hormones were differentially associated with birth outcomes. Biomarkers of EED may be useful to identify pregnant women at risk of adverse birth outcomes, but further research is needed to confirm these findings and elucidate biological mechanisms.

**Funding:**

10.13039/100000002National Institutes of Health.

**Research in context**

**Evidence before this study**

Women living with HIV have a higher risk of adverse birth outcomes, although the reasons for this are not fully understood. Environmental enteric dysfunction (EED), a subclinical condition of the small intestine resulting in increased gut permeability, nutrient malabsorption, gut and systemic inflammation, and growth hormone resistance, may increase the risk of adverse birth outcomes. Very few studies have examined biomarkers of any of these domains among women living with HIV during pregnancy, particularly in sub-Saharan Africa where EED is widespread.

**Added value of this study**

In this prospective cohort study among 706 women living with HIV in Dar es Salaam, Tanzania, we examined the associations of maternal biomarkers of EED, systemic inflammation, and growth hormone resistance with birthweight, birthweight-for-gestational age, and gestation duration. In secondary analyses, we examined associations between these biomarkers with low birthweight, small-for-gestational age, preterm birth, and stillbirth. Although biomarkers of EED were not associated with our primary outcomes, increased maternal intestinal fatty acid-binding protein (I-FABP), a marker of gut epithelial barrier integrity, was associated with increased risk of stillbirth. Furthermore, increased AGP, a marker of systemic inflammation, was associated with lower birthweight and increased risk of small-for-gestational age births. Growth hormones related to growth promotion and regulation were also associated with birthweight and birthweight-for-gestational age, as well as gestation duration and preterm birth. This study examines these multiple domains of EED among women living with HIV and their associations with birth outcomes.

**Implications of all the available evidence**

Maternal biomarkers of EED, systemic inflammation, and the growth hormone axis were differentially associated with adverse birth outcomes, and the relationships were not consistent among biomarkers within these respective domains. Some of the biomarkers examined may be useful to identify pregnant women at risk of adverse birth outcomes and could play a role in the design and evaluation of interventions that address EED, inflammation, and promote healthy pregnancies to improve birth outcomes. Further studies are needed to confirm these results and elucidate the potentially different biological mechanisms involved.

Alt-text: Unlabelled box

## Introduction

Adverse birth outcomes, such as stillbirth, low birth weight (LBW), small for their gestational age (SGA), and preterm birth remain major health concerns in low-income and middle-income countries (LMICs).^1^^,^^2^ Infants who are born LBW, SGA, or premature are known to experience a higher risk of morbidity and mortality,^3^ and they are less likely to attain their full developmental potential.^4^ The risk of adverse birth outcomes is higher as compared to HIV-negative women even for those on antiretroviral therapy (ART).^5^ In 2020, there were approximately 1·3 million live births to pregnant women living with human immunodeficiency virus (PWLHIV).^6^

The increased metabolic needs of pregnancy are accompanied by morphological changes to the gastrointestinal tract such as villus hypertrophy.^7^ Gastrointestinal function, however, has not been widely studied during pregnancy. Though there is a growing body of literature that suggests associations between infections, inflammation, and adverse birth outcomes among PWLHIV,^8^ there are few studies that have examined the potential contribution of enteric dysfunction per se. Environmental enteric dysfunction (EED) is a subclinical condition of the small intestine resulting in increased gut permeability, nutrient malabsorption, and gut and systemic inflammation, likely due to chronic enteropathogen exposure and unhygienic environments.^9^ EED has been proposed as a cause of poor child growth in LMICs,^10^ and the condition also occurs among adults and closely resembles features of HIV enteropathy.^11^^,^^12^ Furthermore, there is evidence that characteristics of EED, such as lower villous height, higher crypt depth, inflammation of the lamina propria, and biomarkers of microbial translocation, are more common among people living with HIV, even on ART, as compared to HIV-negative individuals.^13^^,^^14^

Limited studies have examined the relationship between EED biomarkers and birth outcomes among PWLHIV. Small case-control studies among PWLHIV in Spain and India have reported that increased lipopolysaccharide-binding protein (LBP), soluble CD14 (sCD14), soluble CD163 (sCD163), and intestinal-fatty acid binding protein (I-FABP) were associated with increased risk of preterm birth,^15^^,^^16^ and a small cohort study in Malawi found increased sCD14 was associated with lower birthweight.^17^ However, a recent cohort study in India among pregnant women with and without HIV that examined these same biomarkers (sCD14, CD163, and I-FABP) did not find associations with low birthweight or preterm birth, either among all women regardless of HIV status or among women living with HIV.^18^ To our knowledge, no studies have examined the relationship of biomarkers of EED among PWLHIV and stillbirth, an important yet understudied adverse birth outcome.^1^

One of the features of EED is systemic inflammation,^10^^,^^19^ which is associated with adverse birth outcomes,^20^^,^^21^ including among PWLHIV.^18^ Systemic inflammation also alters the growth hormone axis which affects growth promotion and regulation.^22^ Studies have shown that the growth hormone axis is involved with placental function and prenatal growth.^23^^,^^24^ Though animal and human studies have found associations between markers of growth hormones in amniotic fluid or the placenta and adverse birth outcomes,^21^^,^^24^ there is mixed evidence of the role of circulating maternal growth hormone concentrations.^25^^,^^26^ To the best of our knowledge, no prior studies have assessed the relationship between maternal biomarkers of the growth hormone axis with adverse pregnancy outcomes among PWLHIV.

The possible roles of maternal EED, systemic inflammation, and growth hormone resistance on poor birth outcomes have not been well studied, especially among PWLHIV in LMIC settings. Therefore, we evaluated the association of biomarkers of EED, systemic inflammation, and the growth hormone axis at 32 weeks gestation with birth outcomes among PWLHIV in Dar es Salaam, Tanzania.

## Methods

### Study design

We conducted a prospective cohort study of participants who were enrolled in a randomized, parallel-group, triple-blind placebo-controlled trial of vitamin D_3_ supplementation for pregnant women living with HIV in urban Dar es Salaam, Tanzania. (ClinicalTrials.gov identifier NCT02305927).^27^^,^^28^ The trial was conducted from five public antenatal care clinics that provided prevention of mother to child transmission of HIV (PMTCT) for PWLHIV. During the time of the study, the HIV treatment program implemented the Option B+ approach whereby all PWLHIV were initiated on life-long ART, regardless of immunologic or HIV disease status. Women were eligible for enrollment into the parent trial if they were (i) at least 18 years of age, (ii) pregnant and in the second trimester (12-27 weeks gestation) according to the last menstrual period (LMP) at the time of randomization, (iii) living with HIV, (iv) started ART or had started before the pregnancy, and (v) at the time of screening had serum albumin-adjusted calcium levels in the normal range (<2·6 mmol/L). Exclusion criteria included (i) intention to leave the study area within 2 years after study enrollment, (ii) enrollment in another clinical trial, (iii) refusal to provide informed consent. After study enrollment, pregnant women attended monthly study visits. At 32 weeks gestation, all pregnant women also had a blood draw and had serum samples stored at -80°C in a biorepository.

Using SAS (PROC SURVEYSELECT, version 9.4), we randomly selected 725 participants, using a simple random sample of all 2300 participants enrolled in the trial. Participants were eligible for the EED study and assessment of the panel of biomarkers if they had a serum sample available at the 32-week gestation visit. Based on our calculation, a sample size of 600 is needed to have a 90% power to detect an effect size of 0·018 (that is, 1·8% of the variance in the outcome attributed to a given biomarker), with significance level (alpha) of 0·05, one tested predictor, and ten additional independent covariates. We increased our sample size to 725 to account for a possible 17% loss to follow-up. We additionally restricted analysis to singleton births.

### Laboratory analyses

All samples selected for the cohort study were to have the full panel of EED, systemic inflammation and growth hormone biomarkers quantified. ELISA methods were used to measure anti-flagellin and anti-LPS Ig concentrations (IgA and IgG) according to previously reported methods.^29^ Briefly, microtiter plates were coated with purified *Escherichia coli* LPS (1 µg/well) or *Escherichia coli* flagellin (100 ng/well). Serum samples were diluted (1:500) and then applied to wells coated with LPS or flagellin, followed by incubation and washing. Next, wells were incubated with anti-IgA (KPL, Catalog No. 14-10-01) or anti-IgG (GE, Catalog No. 375112) coupled to horseradish peroxidase. Total immunoglobulins were quantitated via colorimetric peroxidase substrate tetramethylbenzidine using an ELISA plate reader. Samples were read at 450nm and 540nm, and concentrations are reported as optical density (OD) units corrected for background value blanks. Serum samples were also processed at Quansys Biosciences (Logan, UT), on the Q-Plex™ Human Environmental Enteric Dysfunction multiplexed ELISA array, also known as the Micronutrient and EED Assessment Tool (MEEDAT). MEEDAT measures two indicators of EED: I-FABP which is indicative of gut epithelial barrier integrity (damage/repair) as well as sCD14 which is indicative of monocyte activation and is a marker of microbial translocation through the intestinal epithelial barrier. MEEDAT additionally measures acute phase systemic inflammation (α1- acid glycoprotein [AGP], C-reactive protein [CRP]), as well as hormones involved in the insulin-like growth hormone axis (insulin-like growth factor 1 [IGF-1] and fibroblast growth factor 21 [FGF21]). Per the manufacturer's recommendation, a serum dilution of 1:10 was used. Quantitative concentrations were calculated, and the lot lower limit of quantification (LLOQ) and the lot upper limit of quantification (ULOQ) were calculated using calibrator curves. All values below the lot lower limit of detection (LLOD) were substituted with a value of half the value of the LLOQ, and all values between the LLOD and LLOQ were extrapolated using calibrator curves, as were values above the ULOQ. All values that were incalculably high (values above calibrator curve) were included in quartile analyses but excluded from secondary log_2_ analyses. The intra-assay precision for anti-flagellin IgA and IgG and anti-LPS IgA and IgG was 9.2%, 11.4%, 9.8%, and 12.5% respectively. The intra-assay precision for sCD14 was 4%, I-FABP was 6%, for CRP was 3%, for AGP was 5%, IGF-1 was 7%, and for FGF21 was 4%. Detailed laboratory methods used for the MEEDAT panel are found in Appendix 1, and information about its validation has been previously published.^30^

### Outcomes

The primary outcomes of the study were birthweight in grams, gestational duration in weeks, and birthweight for gestational age z-scores. Study nurses and midwives attending the labour and delivery assessed birthweight among participants giving birth in Dar es Salaam. Birthweights for deliveries at study facilities were measured to the nearest 5 g using a digital scale (SECA, Hamburg, Germany). Information on deliveries outside of Dar es Salaam was obtained from medical records and attending clinical staff (∼10% of deliveries). LMP dating was used to determine gestation duration. Birthweight for gestational age z-scores were determined according to INTERGROWTH-21^st^ standards.^31^ We also examined low birthweight (<2500 g), preterm birth (<37·0 weeks completed gestation), and small-for-gestational age (SGA). SGA was defined as <10^th^ percentile birthweight for gestational age by sex according to INTERGROWTH-21^st^ standards.^31^

### Statistical analysis

Biomarkers were categorized into quartiles, with the lowest quartile as the reference group. Linear regression models with robust standard errors to address deviations from normality were used to assess the relationship between biomarker quartiles and continuous outcomes. Log-binomial regression models were used to assess the relative risk of binomial outcomes by biomarker quartiles.^32^ If the log-binomial models did not converge, log-Poisson models, which provide consistent but not fully efficient estimates of the relative risk and its confidence intervals were used.^33^ The P-value for trend in quartile analyses was calculated by regressing the median value of each quartile on the outcome of interest. As secondary analyses, we analysed the biomarkers as continuous variables with log_2_ transformations. In these models, each unit increase in log_2_ concentration is interpreted as a doubling of the biomarker concentration.

Multivariable models included adjustment for maternal age (18-24, 25-34, 35+ years), body mass index (BMI) (<18·5 kg/m^2^, 18·5-24·9 kg/m^2^, 25·0-29·9 kg/m^2^, ≥30 kg/m^2^), marital status (married/cohabitating, single, widowed/married/divorced), education (no formal education completed, completed primary, completed primary or higher), socioeconomic status (SES), clinic site, World Health Organization (WHO) HIV disease stage (I, II, III or IV), CD4 T-cell count (<200, 200-500, >500 cells per μL), the timing of ART initiation (before vs during pregnancy), infant sex (male vs female), and randomized regimen (vitamin D_3_ vs. placebo). SES was defined according to household ownership of assets (electricity, couch, television, refrigerator, fan, bicycle, and car), and was categorized into quintiles using a principal components analysis. We also conducted sensitivity analysis that adjusted for CRP to address the potential contribution of systemic inflammation. The missing indicator method was used to retain individuals with missing covariates when a missing covariate applied to more than 1% of participants, which applied to maternal BMI (1.1% missing) and CD4 count (47.0% missing). In order to assess the robustness of the missing indicator method, we conducted sensitivity analyses using multiple imputation with chained equations to impute missing covariate data for maternal BMI (1.1% missing) and CD4 T-cell count (47.0% missing). Early during the conduct of the trial, the HIV care and treatment program discontinued routine CD4 T-cell assessment after ART initiation, and switched to only HIV-1 viral load assessments every 6 months of ART treatment. The trial relied on the program for CD4 counts and therefore after programmatic discontinuation, CD4 counts are missing for the later trial enrollments. Thus, CD4 counts were not available for all participants. All analyses were conducted in STATA 15 (College Station, Texas, USA).

### Ethics

The parent trial protocol was approved by the Harvard T.H. Chan School of Public Health Institutional Review Board (reference no. IRB14-3416), the Tanzanian National Health Research Ethics Sub-Committee (NatHREC; reference no. NIMR/HQ/R.8a/Vol.IX/1933), the Muhimbili University of Health and Allied Science Institutional Review Board (2016-05-25/AEC/Vol.X/01) and the Tanzania Food and Drug Authority (TFDA; reference no. TFDA15/CTR/0003/5). All participants provided written informed consent for collection of blood and storage of samples for future studies in the parent trial. This ancillary study was approved by Boston Children's Hospital [IRB- P00033836), HSPH (IRB20-1662), Tanzanian National Health Research Ethics Sub-Committee (NIMR/HQ/R.8a/Vol. IX/3309), and the Muhimbili University of Health and Allied Science Institutional Review Board (MUHAS-REC-11-2019-065).

### Role of the funding source

The funders had no role in the study design, data collection and analysis, decision to publish, or preparation of the manuscript.

## Results

**Figure 1:**
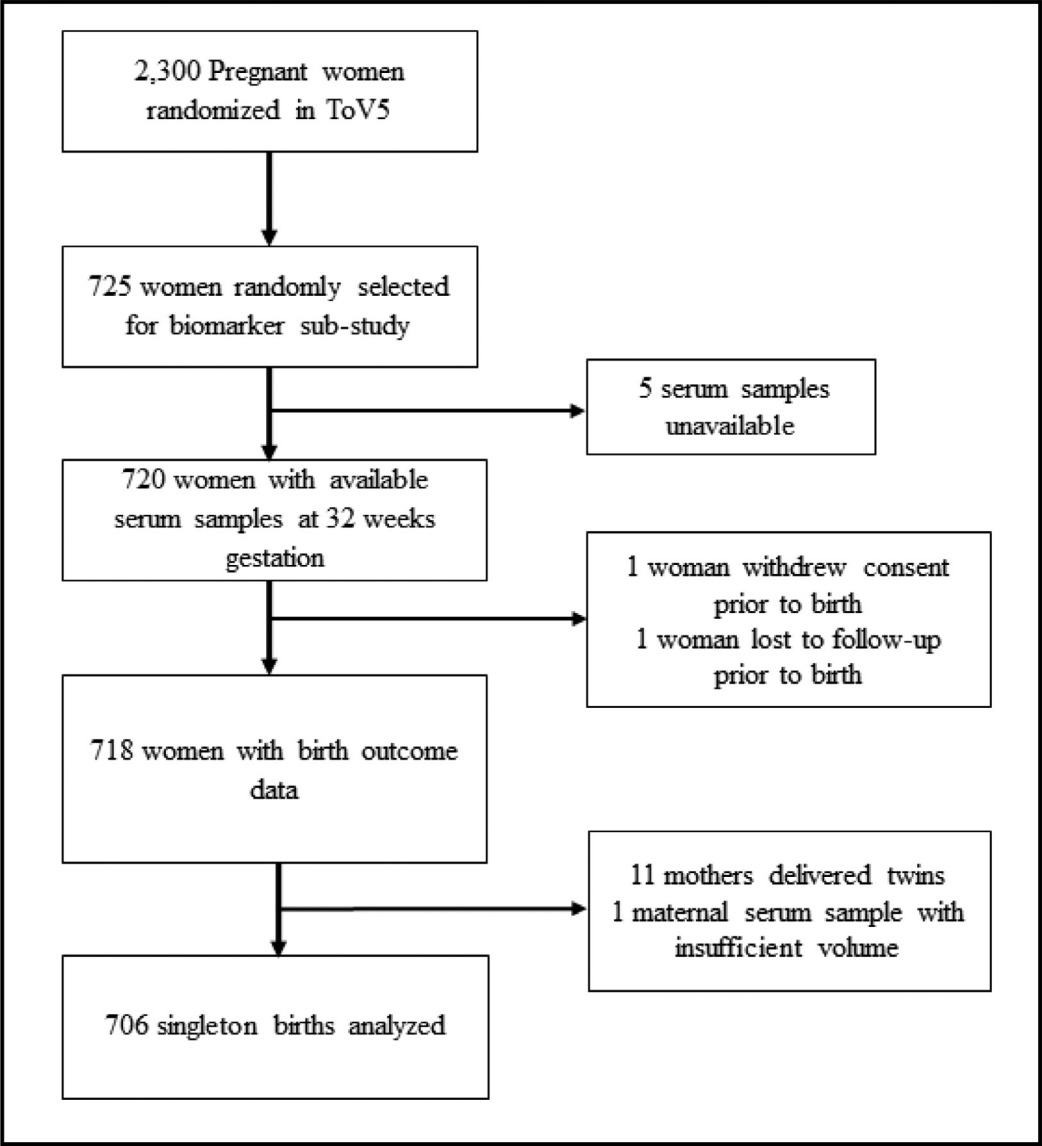
Participant flow chart of pregnant women living with HIV in Dar es Salaam, Tanzania included in the analysis.

The flow diagram for participants and sample selection with the resulting study population included in the analysis is presented in Figure 1. Among the 725 mothers who were randomly selected from the parent vitamin D supplementation trial, 720 had serum samples available for analysis. Of those with available serum samples, one participant withdrew consent, and one participant was lost to follow-up before birth outcomes were ascertained. Eleven participants delivered twins and were excluded from analysis, and one participant had insufficient serum volume for analysis. Therefore, the analytic population included samples from 706 PWLHIV.

Baseline characteristics of the study population are shown in Table 1. The mean maternal age was 30·6 years (SD 5·6 yrs), and the mean BMI was 25·8 kg/m^2^ (± 4·9 kg/m^2^) at baseline. The first-line ART regimen for 99% of enrolled women was Tenofovir/Lamivudine/Efavirenz (Table 1). Baseline factors of mothers included in the analysis were generally similar on all factors as compared to those that were not included in the analysis, except for a small but significant difference in CD4 count – those included in the analysis were slightly less likely to have a missing CD4 count (Table S1). Among the 706 singleton births that were analysed, there were 32 stillbirths (4·5%). The mean birthweight was 3150g (SD 485g), mean gestational age at delivery was 39·3 weeks (SD 2·4 weeks), and mean birthweight –for-gestational age z-score was -0·13 (SD 1·27) (Table S2). Among the 674 live births, 41 were LBW (6·1%), 96 were preterm (14·2%), and 127 were SGA (18·8%). At 6 weeks of age 0.4% (3/674) of children tested positive for HIV. Biomarker levels (OD units and concentrations as applicable) are presented in Table 2, details about biomarker limits of detection and quantification are in Table S3, and Pearson correlations between biomarkers are presented in Table S4.

**Table 1:** Baseline characteristics of pregnant women living with HIV included in the analysis (n=706).

Mother characteristics	N (%) or mean (SD)
Age (n=706)	
18–24	105 (14·9)
25–34	415 (58·8)
35+	186 (26·3)
Maternal Education (n=705)	
No formal education completed	82 (11·6)
Completed primary	391 (55·5)
Completed secondary or higher	232 (32·9)
Marital status (n=705)	
Married/cohabitating	541 (76·7)
Single	145 (20·6)
Widowed / divorced / separated	19 (2·7)
Body mass index (n=698)	
<18·5 kg/m^2^	20 (2·9)
18·5-24·9 kg/m^2^	318 (45·6)
25·0-29·9 kg/m^2^	228 (32·7)
≥30·0 kg/m^2^	132 (18·9)
Parity (n=706)	
1	173 (24·5)
2-3	402 (56·9)
≥4	131 (18·6)
WHO HIV disease stage (n=706)	
I	603 (85·4)
II	45 (6·4)
III or IV	58 (8·2)
CD4 T-cell count, cells per µL (n=374)	
<200	83 (22·2)
200-500	210 (56·1)
>500	81 (21·7)
Timing of antiretroviral therapy (ART) initiation (n=706)	
During this pregnancy	412 (58·4)
Before conception	294 (41·6)
ART regimen (n=700)	
Tenofovir/Lamivudine/Efavirenz	698 (99·7)
Other	2 (0·3)
Regimen (n=706)	
Placebo	363 (51·4)
Vitamin D_3_	343 (48·6)

**Table 2:** Maternal biomarkers at 32 weeks gestation among women living with HIV in Dar es Salaam, Tanzania.

Maternal biomarkers (32 weeks gestation)	n	Mean (SD)	Median (IQR)
Anti-Flagellin IgA (OD)	705	1·63 (0·68)	1·60 (1·11-2·09)
Anti-Flagellin IgG (OD)	705	1·75 (0·52)	1·75 (1·39-2·13)
Anti-LPS IgA (OD)	705	1·57 (0·75)	1·47 (1·00-2·05)
Anti-LPS IgG (OD)	705	1·83 (0·63)	1·80 (1·33-2·34)
sCD14 (ng/mL)	706	3910·89 (2290·58)	3271·15 (2362·49-5002·02)
I-FABP (pg/mL)	706	1650·86 (1210·25)	1348·11 (737·37-2280·52)
AGP (g/L)	706	1·10 (0·74)	0·92 (0·68-1·30)
CRP (mg/L)	706	15·83 (15·35)	11·47 (5·12-20·91)
IGF-1 (ng/mL)	706	186·31 (127·09)	170·03 (95·370- 250·35)
FGF21 (pg/mL)	706	1027·98 (1151·81)	575·79 (245·19- 1448·06)

OD, optical density; IgA, immunoglobulin A; IgG, immunoglobulin G; LPS, lipopolysaccharide; sCD14, soluble CD14; I-FABP, intestinal fatty acid-binding protein; CRP, C-reactive protein; AGP, α1-acid glycoprotein; IGF-1, insulin-like growth factor 1; FGF21, fibroblast growth factor 21 (FGF21); ng, nanograms; pg, picograms; mg, milligrams; mL, milliliters; L, liter; SD, standard deviation; IQR, interquartile range.

### Biomarkers of EED

We examined the associations between quartiles of EED biomarkers (anti-LPS Igs, anti-flagellin Igs, sCD14, I-FABP) and birth outcomes in univariable (Table S5) and multivariable analyses (Table 3). In multivariable analyses, there were no significant associations between these biomarkers of maternal EED and birthweight, gestational duration, or birthweight-for gestational age (Table 3), nor LBW, SGA, or preterm birth in univariable (Table S6) and multivariable analyses (Table 4). In both univariable and multivariable analysis, EED biomarkers analysed continuously as log_2_-transformed continuous exposures had no significant associations with birthweight, gestational duration, or birthweight-for gestational age (Table S9), nor LBW, SGA, or preterm birth (Table S10). In sensitivity analyses, maternal EED biomarker quartiles nor continuous values were associated with birth outcomes when also adjusting for CRP (Tables S7, S8, S11, and S12).

**Table 3:** Multivariable association of analyte concentrations (optical density or concentration in quartiles) at 32 weeks gestation with infant birthweight, birthweight-for-gestational age z-score, and gestational age at birth, among 674 women living with HIV in Dar es Salaam Tanzania.^a^

	Birthweight (g)				Birthweight-for-gestational age (z-score)				Gestational age (weeks)			
	Mean (SD)	Mean diff (g)	95% CI	*p*	Mean (SD)	Mean diff (z-score)	95% CI	*p*	Mean (SD)	Mean diff (wks)	95% CI	*p*
**Biomarkers of EED**												
Flagellin IgG (OD)				0·53				0·94				0·41
1 (0·087-1·385)	3129 (449)	Ref			−0·24 (1·27)	Ref			39·5 (2·4)	Ref		
2 (1·387-1·748)	3166 (471)	66	(−33, 164)		0·01 (1·21)	0·29	(0·02, 0·56)		39·1 (2·3)	−0·4	(−0·9, 0·1)	
3 (1·751-2·126)	3186 (526)	48	(−52, 147)		−0·12 (1·36)	0·10	(−0·18, 0·37)		39·5 (2·4)	0·0	(−0·5, 0·5)	
4 (2·128-3·336)	3116 (494)	−29	(−125, 67)		−0·15 (1·25)	0·06	(−0·21, 0·33)		39·2 (2·5)	−0·3	(−0·9, 0·2)	
Flagellin IgA (OD)				0·07				0·44				0·13
1 (0·040-1·114)	3218 (464)	Ref			−0·09 (1·28)	Ref			39·7 (2·4)	Ref		
2 (1·121-1·604)	3137 (513)	−65	(−168, 37)		−0·09 (1·27)	−0·00	(−0·28, 0·27)		39·2 (2·4)	−0·5	(−1·0, 0·1)	
3 (1·607-2·092)	3123 (485)	−84	(−185, 17)		−0·11 (1·29)	0·01	(−0·27, 0·29)		39·1 (2·4)	−0·7	(−1·2, -0·1)	
4 (2·097-3·259)	3117 (475)	−93	(−193, 7)		−0·22 (1·26)	−0·11	(−0·39, 0·17)		39·4 (2·3)	−0·4	(−0·9, 0·1)	
LPS IgG (OD)				0·71				0·96				0·63
1 (0·089-1·328)	3138 (494)	Ref			−0·19 (1·34)	Ref			39·5 (2·5)	Ref		
2 (1·329-1·802)	3164 (464)	19	(−82, 121)		−0·04 (1·19)	0·11	(−0·16, 0·38)		39·2 (2·2)	−0·2	(−0·8, 0·3)	
3 (1·803-2·342)	3149 (487)	16	(−84, 116)		−0·14 (1·23)	0·05	(−0·22, 0·32)		39·4 (2·3)	−0·1	(−0·6, 0·4)	
4 (2·346-3·367)	3146 (499)	−18	(−119, 83)		−0·13 (1·34)	0·01	(−0·27, 0·30)		39·3 (2·5)	−0·2	(−0·7, 0·3)	
LPS IgA(OD)				0·27				0·74				0·14
1 (0·017-1·005)	3162 (479)	Ref			−0·22 (1·29)	Ref			39·7 (2·3)	Ref		
2 (1·010-1·465)	3156 (503)	−5	(−107, 98)		0·00 (1·28)	0·22	(−0·06, 0·49)		39·1 (2·7)	−0·6	(−1·1, -0·0)	
3 (1·468-2·054)	3189 (492)	20	(−76, 116)		−0·02 (1·24)	0·18	(−0·09, 0·44)		39·2 (2·3)	−0·4	(−0·9, 0·1)	
4 (2·054-3·441)	3088 (465)	−59	(−156, 38)		−0·26 (1·26)	−0·01	(−0·28, 0·26)		39·3 (2·1)	−0·5	(−0·9, 0·0)	
sCD14 (ng/mL)				0·52				0·50				0·72
1 (16·770-2362·485)	3150 (489)	Ref			−0·10 (1·24)	Ref			39·3 (2·4)	Ref		
2 (2365·170-3270·630)	3133 (455)	−35	(−135, 65)		−0·21 (1·27)	−0·14	(−0·41, 0·13)		39·4 (2·3)	0·1	(−0·4, 0·6)	
3 (3271·670-5002·015)	3157 (492)	−0	(−104, 103)		−0·12 (1·28)	−0·03	(−0·30, 0·23)		39·4 (2·5)	0·1	(−0·4, 0·6)	
4 (5005·980-19607·785)	3158 (507)	20	(−82, 122)		−0·07 (1·31)	0·04	(−0·22, 0·31)		39·2 (2·4)	−0·0	(−0·5, 0·4)	
I-FABP (pg/mL)				0·96				0·79				0·58
1 (1·385-737·365)	3145 (447)	Ref			−0·15 (1·31)	Ref			39·4 (2·2)	Ref		
2 (739·435-1346·800)	3187 (488)	60	(−38, 159)		−0·06 (1·21)	0·14	(−0·13, 0·41)		39·5 (2·5)	0·1	(−0·4, 0·6)	
3 (1349·415- 2280·515)	3111 (494)	−9	(−105, 87)		−0·18 (1·26)	0·00	(−0·26, 0·27)		39·3 (2·3)	−0·1	(−0·6, 0·4)	
4 (2280·515- 8338·955)	3156 (512)	25	(−77, 126)		−0·11 (1·33)	0·08	(−0·19, 0·36)		39·3 (2·4)	−0·1	(−0·6, 0·4)	
**Systemic inflammation**												
AGP (g/L)				0·01				0·09				0·23
1 (0·180-0·680)	3233 (492)	Ref			0·05 (1·32)	Ref			39·5 (2·6)	Ref		
2 (0·685-0·920)	3134 (474)	−122	(−223, -21)		−0·17 (1·18)	−0·27	(−0·54, 0·00)		39·3 (2·3)	−0·1	(−0·7, 0·4)	
3 (0·925-1·295)	3160 (467)	−62	(−161,37)		−0·13 (1·19)	−0·18	(−0·44, 0·09)		39·4 (2·2)	−0·0	(−0·5, 0·4)	
4 (1·300-8·040)	3065 (497)	−163	(−269, -57)		−0·27 (1·39)	−0·31	(−0·60, -0·01)		39·2 (2·4)	−0·3	(−0·9, 0·2)	
CRP (mg/L)				0·26				0·81				0·24
1 (0·245-5·115)	3145 (504)	Ref			−0·17 (1·27)	Ref			39·5 (2·5)	Ref		
2 (5·150-11·460)	3196 (450)	25	(−74, 124)		−0·03 (1·24)	0·10	(−0·16, 0·37)		39·4 (2·3)	−0·2	(−0·7, 0·3)	
3 (11·480- 20·910)	3133 (495)	−27	(−129, 76)		−0·15 (1·28)	0·00	(−0·27, 0·27)		39·3 (2·3)	−0·3	(−0·7, 0·2)	
4 (20·915-115·020)	3123 (490)	−42	(−144, 61)		−0·16 (1·30)	0·01	(−0·26, 0·28)		39·2 (2·5)	−0·4	(−0·9, 0·2)	
**Growth hormone axis**												
IGF-1 (ng/mL)				0·003				0·01				0·97
1 (0·375-95·535)	3066 (503)	Ref			−0·34 (1·31)	Ref			39·5 (2·6)	Ref		
2 (96·445-170·920)	3140 (440)	75	(−23, 174)		−0·10 (1·17)	0·25	(−0·02, 0·51)		39·2 (2·3)	−0·3	(−0·8, 0·2)	
3 (171·065-253·650)	3158 (462)	93	(−9, 195)		−0·15 (1·27)	0·19	(−0·09, 0·47)		39·5 (2·3)	0·0	(−0·5, 0·5)	
4 (253·985-1144·363)	3233 (520)	172	(64, 281)		0·08 (1·31)	0·42	(0·14, 0·70)		39·3 (2·3)	−0·1	(−0·7, 0·4)	
FGF21 (pg/mL)				0·09				0·92				0·03
1 (0·465-245·185)	3220 (503)	Ref			−0·08 (1·26)	Ref			39·6 (2·2)	Ref		
2 (246·975-575·790)	3150 (444)	−53	(−151, 44)		−0·13 (1·15)	−0·03	(−0·28, 0·23)		39·4 (2·5)	−0·2	(−0·7, 0·3)	
3 (576·350-1465·635)	3114 (477)	−92	(−194, 9)		−0·22 (1·27)	−0·12	(−0·39, 0·15)		39·3 (2·2)	−0·3	(−0·8, 0·2)	
4 (1468·845-9228·637)	3114 (510)	−101	(−204, 3)		−0·08 (1·41)	0·00	(−0·28, 0·28)		39·0 (2·6)	−0·6	(−1·1, -0·1)	

a Models adjusted for maternal age, maternal BMI, maternal marital status, maternal education, parity, SES, clinic site, WHO HIV stage, CD4 T-cell count, timing of ART initiation, infant sex, and parent trial regimen. The p-values were calculated from linear regression models. OD, optical density; IgA, immunoglobulin A; IgG, immunoglobulin G; LPS, lipopolysaccharide; sCD14, soluble CD14; I-FABP, intestinal fatty acid-binding protein; CRP, C-reactive protein; AGP, α1-acid glycoprotein; IGF-1, insulin-like growth factor 1; FGF21, fibroblast growth factor 21 (FGF21); ng, nanograms; pg, picograms; mg, milligrams; mL, milliliters; L, liters; SD, standard deviation.

**Table 4:** Multivariable association of analyte concentrations (optical density or concentration in quartiles) at 32 weeks gestation with stillbirth, low birthweight (LBW), small-for-gestational age (SGA), and preterm birth, among 706^a^ women living with HIV in Dar es Salaam Tanzania.^b^

	Stillbirth				LBW				SGA				Preterm birth			
	Events, n	RR	95% CI	*p*	Events, n	RR	95% CI	*p*	Events, n	RR	95% CI	*p*	Events, n	RR	95% CI	*p*
**Biomarkers of EED**																
Flagellin IgG (OD)				0·55				0·86				0·48				0·58
1 (0·087-1·385)	5/177	Ref			11/172	Ref			35/172	Ref			23/172	Ref		
2 (1·387-1·748)	10/176	1·91	(0·69, 5·30)		8/166	0·55	(0·21, 1·42)		22/166	0·59	(0·35, 0·99)		24/166	1·06	(0·62, 1·83)	
3 (1·751-2·126)	9/176	1·74	(0·62, 4·83)		11/167	0·86	(0·36, 2·05)		35/167	1·04	(0·68, 1·58)		20/167	0·86	(0·49, 1·52)	
4 (2·128-3·336)	8/176	1·51	(0·48, 4·71)		11/168	0·95	(0·40, 2·25)		35/168	1·04	(0·68, 1·57)		29/168	1·22	(0·73, 2·03)	
Flagellin IgA (OD)				0·17				0·63				0·52				0.24
1 (0·040-1·114)	7/177	Ref			5/170	Ref			30/170	Ref			18/170	Ref		
2 (1·121-1·604)	6/176	0·78	(0·26, 2·31)		14/170	2·79	(0·91, 8·53)		31/170	1·08	(0·68, 1·71)		25/170	1·36	(0·75, 2·46)	
3 (1·607-2·092)	6/176	0·84	(0·29, 2·43)		14/170	3·15	(1·04, 9·52)		31/170	1·05	(0·66, 1·67)		29/170	1·68	(0·96, 2·95)	
4 (2·097-3·259)	13/176	1·67	(0·70, 3·98)		8/163	1·56	(0·47, 5·18)		35/163	1·17	(0·75, 1·81)		24/163	1·37	(0·75, 2·51)	
LPS IgG (OD)				0·26				0·55				0·71				0.27
1 (0·089-1·328)	7/177	Ref			13/170	Ref			35/170	Ref			21/170	Ref		
2 (1·329-1·802)	12/176	1·30	(0·53, 3·19)		8/164	0·61	(0·23, 1·60)		27/164	0·83	(0·52, 1·34)		21/164	0·98	(0·57, 1·71)	
3 (1·803-2·342)	6/176	0·65	(0·23, 1·86)		11/170	0·75	(0·33, 1·71)		30/170	0·85	(0·54, 1·33)		26/170	1·19	(0·69, 2·04)	
4 (2·346-3·367)	7/176	0·71	(0·24, 2·05)		9/169	0·73	(0·31, 1·72)		35/169	1·08	(0·71, 1·64)		28/169	1·29	(0·76, 2·21)	
LPS IgA(OD)				0·07				0·60				0·41				0.51
1 (0·017-1·005)	4/177	Ref			9/173	Ref			35/173	Ref			18/173	Ref		
2 (1·010-1·465)	8/176	2·19	(0·67, 7·19)		13/168	1·64	(0·64, 4·19)		27/168	0·80	(0·51, 1·26)		31/168	1·76	(1·02, 3·04)	
3 (1·468-2·054)	9/176	2·16	(0·69, 6·74)		11/167	1·25	(0·51, 3·06)		26/167	0·77	(0·48, 1·21)		21/167	1·13	(0·62, 2·05)	
4 (2·054-3·441)	11/176	2·83	(0·92, 8·71)		8/165	0·90	(0·33, 2·46)		39/165	1·16	(0·78, 1·71)		26/165	1·46	(0·83, 2·59)	
sCD14 (ng/mL)				0·68				0·29				0·58				0.91
1 (16·770-2362·485)	5/177	Ref			14/172	Ref			29/172	Ref			24/172	Ref		
2 (2365·170-3270.630)	7/176	1·34	(0·44, 4·09)		10/169	0·87	(0·37, 2·01)		33/169	1·24	(0·79, 1·96)		20/169	0·83	(0·48, 1·43)	
3 (3271.670-5002.015)	12/177	2·06	(0·75, 5·60)		8/165	0·56	(0·22, 1·44)		31/165	1·23	(0·77, 1·96)		28/165	1·14	(0·70, 1·88)	
4 (5005.980-19607·785)	8/176	1·33	(0·48, 3·70)		9/168	0·63	(0·26, 1·55)		34/168	1·19	(0·76, 1·87)		24/168	0·92	(0·55, 1·53)	
I-FABP (pg/mL)				0·02				0·74				0·68				0.64
1 (1·385-737.365)	5/177	Ref			10/172	Ref			33/172	Ref			24/172	Ref		
2 (739.435-1346.800)	5/176	1·06	(0·32, 3·55)		8/171	0·76	(0·29, 2·00)		33/171	1·03	(0·66, 1·59)		21/171	0·89	(0·51, 1·54)	
3 (1349.415- 2280.515)	9/177	2·04	(0·75, 5·56)		13/168	1·34	(0·55, 3·26)		31/168	0·97	(0·62, 1·51)		25/168	1·03	(0·61, 1·74)	
4 (2280.515- 8338·955)	13/176	2·44	(1·00, 5·97)		10/163	1·04	(0·39, 2·80)		30/163	0·93	(0·60, 1·45)		26/163	1·07	(0·65, 1·76)	
**Systemic inflammation**																
AGP (g/L)				0·11				0·88				0·03				0.57
1 (0·180-0·680)	7/180	Ref			9/173	Ref			26/173	Ref			26/173	Ref		
2 (0·685-0·920)	4/173	0·58	(0·17, 2·00)		12/169	1·22	(0·47, 3·20)		31/169	1·31	(0·81, 2·12)		23/169	0·84	(0·50, 1·41)	
3 (0·925-1·295)	7/178	0·88	(0·34, 2·31)		10/171	0·98	(0·35, 2·78)		29/171	1·17	(0·72, 1·90)		25/171	0·88	(0·53, 1·46)	
4 (1·300-8·040)	14/175	1·63	(0·66, 4·00)		10/161	1·02	(0·37, 2·79)		41/161	1·69	(1·07, 2·65)		22/161	0·83	(0·49, 1·40)	
CRP (mg/L)				0·20				0·67				0·25				0.73
1 (0·245-5·115)	4/177	Ref			13/173	Ref			35/173	Ref			25/173	Ref		
2 (5·150-11·460)	7/176	1·40	(0·39, 5·01)		7/169	0·61	(0·23, 1·63)		25/169	0·76	(0·47, 1·22)		26/169	1·15	(0·70, 1·91)	
3 (11·480- 20·910)	10/177	1·84	(0·62, 5·40)		12/167	0·94	(0·42, 2·12)		29/167	0·89	(0·56, 1·40)		19/167	0·84	(0·48, 1·49)	
4 (20·915-115·020)	11/176	2·03	(0·67, 6·13)		9/165	0·72	(0·29, 1·80)		38/165	1·14	(0·75, 1·71)		26/165	1·15	(0·69, 1·90)	
**Growth hormone axis**																
IGF-1 (ng/mL)				0·08				0·44				0·14				0.75
1 (0·375-95·535)	10/177	Ref			12/167	Ref			40/167	Ref			23/167	Ref		
2 (96.445-170.920)	7/176	0·67	(0·26, 1·69)		11/169	0·85	(0·36, 2·04)		27/169	0·65	(0·42, 1·02)		26/169	1·14	(0·68, 1·92)	
3 (171·065-253.650)	11/177	1·06	(0·47, 2·40)		8/166	0·62	(0·23, 1·67)		34/166	0·88	(0·58, 1·34)		18/166	0·83	(0·47, 1·45)	
4 (253.985-1144.363)	4/176	0·30	(0·08, 1·08)		10/172	0·69	(0·24, 1·97)		26/172	0·64	(0·40, 1·00)		29/172	1·20	(0·72, 2·00)	
FGF21 (pg/mL)				0·22				0·87				0·10				0.02
1 (0·465-245·185)	5/177	Ref			9/172	Ref			28/172	Ref			19/172	Ref		
2 (246·975-575·790)	9/176	2·20	(0·83, 5·87)		11/167	1·22	(0·51, 2·91)		29/167	1·09	(0·68, 1·73)		19/167	1·04	(0·57, 1·90)	
3 (576·350-1465·635)	8/177	1·59	(0·59, 4·29)		10/169	1·02	(0·39, 2·65)		33/169	1·20	(0·76, 1·89)		23/169	1·20	(0·68, 2·11)	
4 (1468·845-9228.637)	10/176	2·26	(0·89, 5·75)		11/166	1·15	(0·44, 2·97)		37/166	1·42	(0·91, 2·22)		35/166	1·77	(1·04, 3·00)	

a N=706 women for stillbirth outcome; N=674 women for livebirth outcomes (low birthweight [LBW], small-for-gestational age [SGA], and preterm birth).

b Models adjusted for age, BMI, marital status, maternal education, parity, SES, clinic site, WHO HIV stage, CD4 T-cell count, timing of ART initiation, infant sex, and parent trial regimen. The p-values were calculated from log binomial models. OD, optical density; IgA, immunoglobulin A; IgG, immunoglobulin G; LPS, lipopolysaccharide; sCD14, soluble CD14; I-FABP, intestinal fatty acid-binding protein; CRP, C-reactive protein; AGP, α1-acid glycoprotein; IGF-1, insulin-like growth factor 1; FGF21, fibroblast growth factor 21 (FGF21); ng, nanograms; pg, picograms; mg, milligrams; mL, milliliters; L, liters.

In secondary analyses, we found that increased maternal I-FABP quartiles were significantly associated with increased risk of stillbirth (p for trend [p*_trend_*]=0·02; p-value derived from log binomial model). Women in the highest quartile of I-FABP at 32 weeks gestation had more than twice the risk of stillbirth compared to those in the lowest quartile (RR 2·44, 95% CI 1·00-5·97) (Table 4). Correspondingly, each unit increase in log_2_ transformed I-FABP was associated with an increased risk of stillbirth (RR 1·36, 95% CI 1·01-1·85, p=0·04; p-value derived from log binomial model) (Table S10). There was also a trend between quartiles of LPS IgA and stillbirth, but the results were not statistically significant (p*_trend_*=0·07; p-value derived from log binomial model). In continuous analysis, each unit increase in log_2_ transformed LPS IgA was associated with an increased risk of stillbirth (RR 1·52, 95% CI 1·04-2·23, p=0·03; p-value derived from log binomial model) (Table S10). The findings for the relationship of I-FABP and LPS IgA with stillbirth were consistent when additionally adjusting for CRP (Table S8, S12).

### Biomarkers of systemic inflammation

Increases in maternal AGP quartiles were significantly associated with lower infant birthweight (p*_trend_*=0·01; p-value derived from linear regression model) in multivariate analyses. Infants whose mothers were in the highest quartile of AGP weighed 163 g less (95% CI -269 to -57g) on average than infants whose mothers were in the lowest quartile (Table 3). Similarly, increasing quartiles of AGP were associated with increased risk for SGA births (p_trend_= 0·03; p-value derived from log binomial model), with those in the highest quartile having a 69% increase in the risk of SGA compared to the lowest quartile (RR 1·69, 95% CI 1·07-2·65) (Table 4). Increased quartiles of AGP tended to be associated with lower birthweight-for-age z-score, but was not statistically significant (p*_trend_* =0·09; p-value derived from linear regression model). In analyses of continuous AGP, each unit increase in log_2_-transformed AGP was associated with reduced birthweight (mean difference: -60 g, 95% CI -113 to -8, p=0·02; p-value derived from linear regression model) (Table S9). Quartiles and log_2_-transformed (continuous) CRP were not associated with any birth outcome.

### Biomarkers of growth hormone resistance

Increases in maternal IGF-1 were significantly associated with higher birthweight (p*_trend_* =0·003; p-value derived from linear regression model), with mothers in the highest quartile giving birth to infants that weighed 172g more than those in the lowest quartile (95% CI 64 to 281g) (Table 3). Quartiles of maternal IGF-1 were also associated with a greater birthweight-for-gestational age z-score among infants (p*_trend_* =0·01; p-value derived from linear regression model); infants born to mothers in the highest quartile had a 0·4 SD higher z-score compared to those in the lowest quartile of IGF-1 (95% CI 0·1 to 0·7 SD). IGF-1 was not associated with stillbirth, gestation duration, or the risk of preterm birth. These relationships were consistent after adjustment for CRP (Tables S7, S8, S11, and S12).

Increases in quartiles of FGF21 were associated with shorter gestation (p*_trend_* =0·03; p-value derived from linear regression model); those in the highest quartile had a gestational duration 0·6 weeks shorter (95% CI -1·1 to -0·1 weeks) than those in the lowest quartile (Table 3). This relationship was also found when examining FGF21 as a continuous log_2_-transformed exposure (mean difference: -0·1 weeks, 95% CI -0·2 to 0·0 weeks, p=0·009; p-value derived from linear regression model) (Table S9). Increased FGF21 concentrations were also significantly associated with the risk of preterm birth (p*_trend_* = 0·02; p-value derived from log binomial model); women with FGF21 in the highest quartile had a 77% higher risk of delivering a preterm birth as compared to the lowest quartile (RR 1·77, 95% CI 1·04 to 3·00). In continuous analyses, each unit increase in log_2_ transformed FGF21 was associated with 26 gram lower birthweight (95% CI -46 to -6g, p=0·01; p-value derived from linear regression model), an 11% increase in the risk of SGA (RR 1·11, 95% CI 1·01 to 1·22, p=0·03; p-value derived from log binomial model), and a 12% increase in the risk of preterm birth (RR 1·12, 95% CI 1·00-1·26, p=0·047; p-value derived from log binomial model). Adjustment for CRP did not meaningfully change these estimates (S11 and S12).

### Sensitivity analyses for missing covariate data

In sensitivity analyses of continuous outcomes, the results for the relationship between AGP and birthweight, IGF-1 and birthweight, IGF-1 and birthweight-for-gestational age, and FGF21 and gestational age, were qualitatively similar when using multiple imputation to account for missing maternal BMI and CD4 T-cell count covariate data (S13-S16). Similarly, in sensitivity analyses of binary outcomes, the results for the relationship between I-FABP and stillbirth, AGP and SGA, and FGF21 and preterm birth, were qualitatively similar when using multiple imputation to account for missing maternal BMI and CD4 T-cell count covariate data (S17-S19).

## Discussion

In this prospective cohort study of PWLHIV in Tanzania, we found no association of maternal EED biomarkers related to microbial translocation (anti-LPS Igs, anti-flagellin Igs, sCD14) or gut epithelial damage (I-FABP) with our primary outcomes of birthweight, gestation duration, or birthweight for gestational age. However, increased quartiles of maternal I-FABP and greater anti-LPS IgA concentrations, biomarkers of EED (gut epithelial damage and microbial translocation respectively), were associated with increased risk of stillbirth, a secondary outcome. We also found that increased AGP, a marker of chronic systemic inflammation, was associated with reduced birthweight and increased risk for SGA births. Higher IGF-1 was associated with increased birthweight and birthweight-for-age z-score, while higher FGF21 was associated with shorter gestation duration and was associated with increased risk of preterm birth.

Our finding of no significant association of any maternal EED biomarker in pregnancy with birthweight or gestation duration is in contrast to our earlier study in Uganda among HIV-negative women that found that higher levels of anti-flagellin IgG and anti-LPS IgG were associated with shorter gestational length, as well as decreases in birth length.^34^ However, serum samples in the Uganda study were collected at approximately 18 weeks gestation, were in a rural context, and excluded PWLHIV which may contribute to differences in the findings. Biomarkers of microbial translocation and monocyte activation (LPS and sCD14, respectively) detected in cord blood plasma have also been found to be associated with premature birth, birthweight, as well as chorioamnionitis, although the US-based study did not report on maternal HIV status.^35^ Although we did not observe any associations with biomarkers of EED and birth outcomes other than stillbirth, it is possible that the contribution of EED biomarkers to the risk of adverse birth outcomes among PWLHIV may be more limited as compared to HIV-negative women. Among the few studies that evaluated biomarkers of EED among those living with HIV, a study in Spain among 72 women found that sCD14 was elevated compared to those without HIV, and was positively associated with preterm delivery.^15^ Increased levels of sCD14 were also found to be associated with reduced birth weight among 149 women living with HIV in Malawi.^17^ A case-control study conducted in Ethiopia, India, and Uganda found that increased intestinal barrier dysfunction (I-FABP) and microbial translocation/monocyte activation (sCD14 and sCD163) increased odds of preterm birth, but CRP was not associated.^16^ Overall, evidence on the relationship of maternal EED with birth outcomes is mixed, and the strength of the relationships may differ by maternal HIV status.

Our finding of an association between the EED biomarkers of I-FABP and LPS IgA with stillbirth is intriguing. Further, these associations were also observed when controlling for CRP, which suggests that intestinal wall integrity and microbial translocation may contribute to stillbirth independent of systemic inflammation in the study population, although systemic inflammation may also be a mediator between EED and birth outcomes. The root causes of stillbirth can be multifactorial and are often unreported or challenging to determine, but may stem in part from poor nutrition, abnormal angiogenesis, foetal asphyxia/hypoxia, infections, local and systemic inflammatory responses, pre-eclampsia, antepartum haemorrhage, or placental conditions.^1^ Though neither I-FABP nor LPS IgA have been associated with stillbirth in previous human studies to the best of our knowledge, animal models of placental hypoxia have implicated maternal LPS circulation.^36^ I-FABP is also a marker for acute injury, intestinal trauma, or sepsis,^37^ which are also associated with risk of stillbirth and therefore multiple biological mechanisms may be possible. For example, intestinal damage resulting from low level ischemia or inflammation may share common pathways with placental insufficiency.^38^ Increased microbial translocation has also been associated with reduced progesterone levels among pregnant PWLHIV,^39^ which has been associated with preterm birth and may be associated with stillbirth.^40^ Additional studies are needed to confirm our findings and to elucidate the potential mechanisms linking these maternal EED biomarkers to the risk of stillbirth.

We also found that increased AGP concentrations, an indicator of chronic systemic inflammation, were associated with lower birthweight and increased risk of SGA. However, there was no association of CRP, an indicator of acute systemic inflammation, with any birth outcome. Inflammation during pregnancy has been previously linked to adverse pregnancy outcomes.^18^^,^^21^ Maternal inflammation can lead to placental and foetal inflammation impairing vascular development and gestational growth, and infections in pregnancy leading to a heightened immune state can further contribute to adverse birth outcomes.^20^^,^^41^ Our finding is similar to a study in Nepal that found that higher levels of maternal serum AGP, but not CRP, were associated with smaller birth size, including weight, length, head circumference, and chest circumference, suggesting chronic low-grade inflammation may inhibit foetal growth.^42^ Another study in Nepal, which collected maternal blood samples at 32 weeks gestation, also found that increased AGP was associated with preterm delivery and decreased birth weight.^43^ In contrast, a recent study in India among 218 women, 32% of whom were PWLHIV, found no associations between AGP or CRP and low birthweight or preterm birth, either overall or by maternal HIV status.^18^ However, the same study found associations between increased interleukin-1β, another marker of systemic inflammation, and increased odds of preterm birth, both overall and among PWLHIV.^18^ Other studies have also found high levels of CRP early in pregnancy to be associated with increased risk of preterm birth, as well as the risk of preeclampsia and growth restriction.^21^ One reason for inconsistent findings on CRP between studies may be the timing of CRP measurement as well as the role of HIV which is known to contribute to heightened immune activation and inflammatory response even with ART.^13^ Our sample collection in the third trimester at 32 weeks may have precluded an ability to detect associations with CRP since inflammation can be dynamic during pregnancy and adverse impacts may manifest earlier or later in gestation.^44^^,^^45^ Furthermore, CRP has a shorter half-life than AGP and may differentially reflect underlying mechanisms leading to adverse birth outcomes, and thus may in part explain inconsistencies in association with birth outcomes.^46^

Higher maternal IGF-1, a key hormone in the growth hormone-IGF axis, was associated with increased birthweight and birthweight-for-age z-score. This was unsurprising, as IGF-1 is an important promoter of growth and plays a role in placental function and foetal development.^23^^,^^24^ We also found that increased FGF21, another key hormone within the growth factor axis, was associated with shorter gestational duration. FGF21 is thought to be an indicator of protein restriction,^47^ which has been associated with increased risk of preterm birth.^48^ There have been limited studies on maternal FGF21 during pregnancy, with mixed findings on its relationship with preeclampsia, a risk factor for preterm birth.^49^ However, a recent case-control study conducted in China found higher serum FGF21 near the time of birth was associated with increased risk of preeclampsia and early-onset preeclampsia in particular, and higher levels of FGF21 were also associated with elevated mean arterial pressure, diastolic blood pressure, and low-density lipoprotein cholesterol.^50^

Although our study has a number of strengths, including a >99% follow-up rate and examination of multiple domains of EED, systemic inflammation, and the growth hormone axis, there are several limitations. First, given the observational design of the study, we cannot infer causality from these associations, as there may be unmeasured (i.e., use of antibiotics, gestational diabetes, and other unmeasured maternal factors) or residual confounding. Second, LMP dating was used and therefore it is likely that there is some misclassification in gestation duration and preterm birth outcomes; however, it is likely that misreport of LMP is non-differential with respect to the biomarkers evaluated which would therefore attenuate measures of association. Similarly, there may be some degree of measurement error in birthweight, particularly for deliveries outside of study clinics. Future studies could be strengthened through more accurate dating approaches such as early pregnancy ultrasound. We also collected blood samples at 32 weeks gestation and therefore were not able to evaluate the relationship of EED biomarkers earlier in pregnancy with birth outcomes nor the relationship of EED with very preterm birth, miscarriage or other events that occur before 32 weeks gestation. Future studies could also examine these biomarkers at multiple time points during pregnancy (including first and second trimester), and evaluate potential associations with miscarriage and foetal loss earlier in gestation. In addition, we cannot determine whether these biomarkers of EED, inflammation and the growth hormone axis preceded or resulted from adverse events (such as infections, abnormal foetal growth, or foetal death) during gestation. Repeated sampling throughout pregnancy could be informative, along with an examination of a wider array of inflammatory and metabolic markers, as well as the use of intestinal biopsies to confirm the presence and severity of EED. Furthermore, we did not correct for multiple hypothesis testing and cannot rule out the possibility that our findings of associations were due to Type 1 errors (incorrectly rejecting the null hypothesis) due in part to multiple testing. Lastly, this study was among PWLHIV in urban Tanzania, with observed low levels of maternal undernutrition which is characteristic of some urban settings in East Africa. Therefore, while the sample is representative of PWLHIV attending HIV clinics in Dar es Salaam, the findings may not be generalizable to other populations and settings.

In conclusion, maternal biomarkers of EED, systemic inflammation, and the growth hormone axis were differentially associated with adverse birth outcomes, and the relationships were not consistent among biomarkers within these respective domains. Although we hypothesized that biomarkers of EED would be associated with adverse birth outcomes among PWLHIV, we did not find significant associations with birthweight, gestation duration, or birthweight-for-gestational age. However, higher I-FABP, a marker of gut epithelial damage, was associated with increased risk of stillbirth. Furthermore, increased systemic inflammation as measured by AGP was associated with decreased birthweight and was associated with increased risk of small-for-gestational age birth. Lastly, hormones related to growth promotion and regulation were associated with adverse outcomes – higher IGF-1 was associated with increased birthweight and birthweight-for-gestational age, while higher FGF21 was associated with shorter gestation duration and increased risk of preterm birth. Further studies are needed to confirm these results and elucidate the potentially different biological mechanisms involved. Taken together, some of the biomarkers examined may be useful to identify pregnant women at risk of adverse birth outcomes and could play a role in the design and evaluation of interventions that address EED, inflammation, and promote healthy pregnancies to improve birth outcomes.

## Contributors

Conceptualization: Miles A. Kirby, Jacqueline M. Lauer, Wafaie W. Fawzi, Karim P. Manji, Christopher P. Duggan, Christopher R. Sudfeld.

Data curation: Miles A. Kirby, Jacqueline M. Lauer, Christopher R. Sudfeld.

Verification of the underlying data: Miles A. Kirby, Christopher R. Sudfeld.

Formal analysis: Miles A. Kirby, Christopher R. Sudfeld.

Funding acquisition: Jacqueline M. Lauer, Robert K.M. Choy, Michael B. Arndt, Andrew Gewirtz, Christopher R. Sudfeld, Karim P. Manji, Said Aboud, Wafaie W. Fawzi, Christopher P. Duggan.

Investigation: Christopher R. Sudfeld, Jianqun Kou, Andrew Gewirtz, Karim P. Manji, Alfa Muhihi, Said Aboud, Wafaie W. Fawzi, Christopher P. Duggan.

Methodology: Christopher R. Sudfeld, Robert K.M. Choy, Michael B. Arndt, Jianqun Kou, Andrew Gewirtz, Alfa Muhihi, Said Aboud, Nzovu Ulenga, Wafaie W. Fawzi, Karim P. Manji, Christopher P. Duggan.

Project administration: Miles A. Kirby, Christopher R. Sudfeld, Karim P. Manji, Alfa Muhihi, Said Aboud, Wafaie W. Fawzi, Christopher P. Duggan.

Supervision: Christopher R. Sudfeld, Robert K.M. Choy, Karim P. Manji, Alfa Muhihi, Said Aboud, Nzovu Ulenga, Wafaie W. Fawzi, Christopher P. Duggan.

Writing – original draft: Miles A. Kirby, Christopher R. Sudfeld, Christopher P. Duggan.

Writing – review & editing: Miles A. Kirby, Jacqueline M. Lauer, Alfa Muhihi, Nzovu Ulenga, Said Aboud, Enju Liu, Robert K.M. Choy, Michael B. Arndt, Jianqun Kou, Andrew Gewirtz, Wafaie Fawzi, Christopher P. Duggan, Karim P. Manji, Christopher R. Sudfeld.

Decision to submit for publication: Christopher P. Duggan, Karim P. Manji, Christopher R. Sudfeld.

All authors read and approved the final version of the manuscript.

## Data sharing statement

The full dataset cannot be shared publicly because of our study's requirement for ethical approval and data transfer agreement. The deidentified dataset supporting this research may be made available following a submitted request to ghp@hsph.harvard.edu and completion of ethical approval and data transfer agreement from the Tanzania National Institute of Medical Research (http://reims.nimr.or.tz:8010/guides/DTA.pdf).

## Declaration of interests

Christopher Duggan declares royalty payments from UpToDate, Inc. The other authors have declared that no competing interests exist.

## Appendix Supplementary materials

Image, application 1
