# Supplementary material for: Biomarkers of environmental enteric dysfunction and adverse birth outcomes: An observational study among pregnant women living with HIV in Tanzania
Source: eBioMedicine. 2022 Sep 18;84:104257. doi: 10.1016/j.ebiom.2022.104257 (PMC9486615; doi:10.1016/j.ebiom.2022.104257)
Supplement: Supplementary file 1 [file mmc1.docx]

**Supplemental Tables and Appendix**

**Supplemental Table 1. Baseline characteristics of pregnant women living with HIV included in the analysis (n=706) and not included in the analysis from parent study (n=1594)**

|  | **Included in the analysis (N=706)** | **Not included in the analysis (n=1594)** | **p-value (Pearson’s chi-squared test)** |
| --- | --- | --- | --- |
| **Mother characteristics** | **N (%)** |  |  |
| Age | N=706 | N=1594 | p=0·59 |
| 18-24 | 105 (14·9) | 264 (16·6) |  |
| 25-34 | 415 (58·8) | 924 (58·0) |  |
| 35+ | 186 (26·3) | 406 (25·5) |  |
|  |  |  |  |
| Maternal Education | N=705 | N=1591 | p=0·14 |
| No formal education completed | 82 (11·6) | 166 (10·4) |  |
| Completed primary | 391 (55·5) | 953 (59·9) |  |
| Completed secondary or higher | 232 (32·9) | 472 (29·7) |  |
|  |  |  |  |
| Marital status | N=705 | N=1,593 | p=0·72 |
| Married/cohabitating | 541 (76·7) | 1198 (75·2) |  |
| Single | 145 (20·6) | 347 (21·8) |  |
| Widowed / divorced / separated | 19 (2·7) | 48 (3·0) |  |
|  |  |  |  |
| Body mass index | N=706 | N=1594 | p=0·79 |
| <18·5 kg/m^2^ | 20 (2·8) | 44 (2·8) |  |
| 18·5-24·9 kg/m^2^ | 318 (45·0) | 723 (45·4) |  |
| 25·0-29·9 kg/m^2^ | 228 (32·3) | 524 (32·9) |  |
| ≥30·0 kg/m^2^ | 132 (18·7) | 293 (18·4) |  |
| Missing | 8 (1·3) | 10 (0·6) |  |
|  |  |  |  |
| Parity | N=706 | N=1594 | p=0·26 |
| 1 | 173 (24·5) | 387 (24·3) |  |
| 2-3 | 402 (56·9) | 953 (59·8) |  |
| ≥4 | 131 (18·6) | 254 (15·9) |  |
|  |  |  |  |
| WHO HIV disease stage | N=706 | N=1594 | p=0·93 |
| I | 603 (85·4) | 1371 (86·0) |  |
| II | 45 (6·4) | 97 (6·1) |  |
| III or IV | 58 (8·2) | 126 (7·9) |  |
|  |  |  |  |
| CD4 T-cell count, cells per µL | N=706 | N=1594 | p<0·001 |
| <200 | 83 (11·8) | 113 (7·1) |  |
| 200-500 | 210 (29·7) | 381 (23·9) |  |
| >500 | 81 (11·5) | 214 (13·4) |  |
| Missing | 332 (47·0) | 886 (55·6) |  |
|  |  |  |  |
| Timing of antiretroviral therapy (ART) initiation | N=706 | N=1594 | p=0·54 |
| During this pregnancy | 412 (58·4) | 642 (40·3) |  |
| Before conception | 294 (41·6) | 952 (59·7) |  |
|  |  |  |  |
| ART regimen | N=700 | N=1589 | p=0·12 |
| Tenofovir/Lamivudine/Efavirenz | 698 (99·7) | 1575 (99·1) |  |
| Other | 2 (0·3) | 14 (0·9) |  |
|  |  |  |  |
| Regimen | N=706 | N=1594 | p=0·40 |
| Placebo | 363 (51·4) | 789 (49·5) |  |
| Vitamin D_3_ | 343 (48·6) | 805 (50·5) |  |

**Supplemental Table 2. Birth outcomes among 706 pregnant women living with HIV in Dar es Salaam, Tanzania participating in biomarker analysis.**

| **Birth characteristics** | Mean (SD) or n (%) |
| --- | --- |
| Stillbirth (≥ 28 weeks gestation) | 32 (4·5) |
| Livebirth | 674 (95·5) |
| *Among livebirths* |  |
| Female | 317 (47·0) |
| Mean birthweight (g) | 3150 (485) |
| Low birth weight (<2500g) | 41 (6·1) |
| Mean gestational age (weeks), livebirths | 39·3 (2·4) |
| Preterm <37 weeks | 96 (14·2) |
| Mean Birthweight for gestational age z-score | -0·13 (1·27) |
| Small-for-gestational age | 127 (18·8) |

**Supplemental Table 3. Distribution of maternal biomarkers at 32 weeks gestation among women living with HIV in Dar es Salaam, Tanzania included in biomarker analysis**

| **Maternal biomarkers (32 weeks gestation)** | **n** | **Mean (SD)** | **Median (IQR)** | **Lot LLOD** | **Below lot LLOD**  **n (%)** | **Lot LLOQ** | **Below lot LLOQ**  **n (%)** | **Lot ULOQ** | **Above lot ULOQ**  **n (%)** | **Incalculable high***  **n (%)** |
| --- | --- | --- | --- | --- | --- | --- | --- | --- | --- | --- |
| Anti-Flagellin IgA (OD) | 705 | 1·63 (0·68) | 1·60 (1·11-2·09) |  |  |  |  |  |  |  |
| Anti-Flagellin IgG (OD) | 705 | 1·75 (0·52) | 1·75 (1·39-2·13) |  |  |  |  |  |  |  |
| Anti-LPS IgA (OD) | 705 | 1·57 (0·75) | 1·47 (1·00-2·05) |  |  |  |  |  |  |  |
| Anti-LPS IgG (OD) | 705 | 1·83 (0·63) | 1·80 (1·33-2·34) |  |  |  |  |  |  |  |
| sCD14 (ng/mL) | 706 | 3910·89 (2290·58) | 3271·15 (2362·49-5002·02) | 1·43 | 0 (0·0) | 3·08 | 2 (0·3) | 2245·82 | 0 (0·0) | 0 (0·0) |
| I-FABP (pg/mL) | 706 | 1650·86 (1210·25) | 1348·11 (737·37-2280·52) | 0·17 | 1 (0·1) | 2·73 | 2 (0·3) | 2147·02 | 0 (0·0) | 0 (0·0) |
| AGP (g/L) | 706 | 1·10 (0·74) | 0·92 (0·68-1·30) | 0·0002 | 0 (0·0) | 0·00042 | 0 (0·0) | 0·31 | 15 (2·1) | 0 (0·0) |
| CRP (mg/L) | 706 | 15·83 (15·35) | 11·47 (5·12-20·91) | 0·0069 | 0 (0·0) | 0·0087 | 0 (0·0) | 5·48 | 22 (3·1) | 0 (0·0) |
| IGF-1 (ng/mL) | 706 | 186·31 (127·09) | 170·03 (95·37- 250·35) | 0·23 | 6 (0·8) | 0·75 | 23 (3·3) | 202·02 | 0 (0·0) | 3 (0·4) |
| FGF21 (pg/mL) | 706 | 1027·98 (1151·81) | 575·79 (245·19- 1448·06) | 0·21 | 1 (0·1) | 0·93 | 5 (0·7) | 688·02 | 3 (0·4) | 1 (0·1) |

OD, optical density; IgA, immunoglobulin A; IgG, immunoglobulin G; LPS, lipopolysaccharide; sCD14, soluble CD14; I-FABP, intestinal fatty acid-binding protein; CRP, C-reactive protein; AGP, α1-acid glycoprotein; IGF-1, insulin-like growth factor 1; FGF21, fibroblast growth factor 21 (FGF21); ng, nanograms; pg, picograms; mg, milligrams; mL, milliliters; L, liters; SD, standard deviation; IQR, interquartile range; LLOD, lower limit of detection; LLOQ, lower limit of quantification; ULOQ, upper limit of quantification

*Included in quartile models but not included in continuous log_2_ models. Excluded from mean, SD, median and IQR descriptive statistics.

**Supplemental Table 4. Pearson correlations of maternal biomarkers at 32 weeks gestation among women living with HIV in Dar es Salaam, Tanzania participating in biomarker analysis**

| Variables | Flagellin IgG | Flagellin IgA | LPS  IgG | LPS IgA | sCD14 | I-FABP | AGP | CRP | IGF-1 | FGF21 |
| --- | --- | --- | --- | --- | --- | --- | --- | --- | --- | --- |
| Flagellin IgG | 1·000 |  |  |  |  |  |  |  |  |  |
| Flagellin IgA | 0·560*** | 1·000 |  |  |  |  |  |  |  |  |
| LPS IgG | 0·768*** | 0·477*** | 1·000 |  |  |  |  |  |  |  |
| LPS IgA | 0·600*** | 0·895*** | 0·628*** | 1·000 |  |  |  |  |  |  |
| sCD14 | 0·048 | -0·007 | 0·010 | 0·013 | 1·000 |  |  |  |  |  |
| I-FABP | 0·006 | -0·005 | -0·026 | 0·011 | 0·494*** | 1·000 |  |  |  |  |
| AGP | 0·087* | 0·058 | 0·047 | 0·062 | 0·534*** | 0·318*** | 1·000 |  |  |  |
| CRP | 0·097* | 0·014 | 0·045 | 0·012 | 0·277*** | 0·179*** | 0·478*** | 1·000 |  |  |
| IGF-1 | -0·039 | -0·063 | -0·036 | -0·025 | 0·438*** | 0·368*** | 0·101** | 0·040 | 1·000 |  |
| FGF21 | 0·034 | -0·001 | 0·012 | 0·012 | 0·501*** | 0·295*** | 0·409*** | 0·161*** | 0·300*** | 1·000 |
| **** p<0·001, ** p<0·01, * p<0·05* | | | | | | | | | | |

IgA, immunoglobulin A; IgG, immunoglobulin G; LPS, lipopolysaccharide; sCD14, soluble CD14; I-FABP, intestinal fatty acid-binding protein; CRP, C-reactive protein; AGP, α1-acid glycoprotein; IGF-1, insulin-like growth factor 1; FGF21, fibroblast growth factor 21

**Supplemental Table 5. Univariable and multivariable association of analyte concentrations (optical density or concentration in quartiles) at 32 weeks gestation with infant birthweight, birthweight-for-gestational age z-score, and gestational age at birth, among women living with HIV in Dar es Salaam Tanzania.^‡^**

|  | Birthweight | | | | | | | Birthweight-for-gestational age z score | | | | | | | Gestational age (weeks) | | | | | | |
| --- | --- | --- | --- | --- | --- | --- | --- | --- | --- | --- | --- | --- | --- | --- | --- | --- | --- | --- | --- | --- | --- |
|  |  | Unadjusted | | | Adjusted | | |  | Unadjusted | | | Adjusted | | |  | Unadjusted | | | Adjusted | | |
|  | Mean (SD) | Mean diff (g) | 95% CI | p | Mean diff (g) | 95% CI | p | Mean (SD) | Mean diff (z-score) | 95% CI | p | Mean diff (z-score) | 95% CI | p | Mean (SD) | Mean diff (wks) | 95% CI | p | Mean diff (wks) | 95% CI | p |
| **Biomarkers of EED** |  |  |  |  |  |  |  |  |  |  |  |  |  |  |  |  |  |  |  |  |  |
| Flagellin IgG (OD) |  |  |  | 0·90 |  |  | 0·53 |  |  |  | 0·67 |  |  | 0·94 |  |  |  | 0·39 |  |  | 0·41 |
| 1 (0·087-1·385) | 3129 (449) | Ref |  |  | Ref |  |  | -0·24 (1·27) | Ref |  |  | Ref |  |  | 39·5 (2·4) | Ref |  |  | Ref |  |  |
| 2 (1·387-1·748) | 3166 (471) | 37 | (-61, 135) |  | 66 | (-33, 164) |  | 0·01 (1·21) | 0·25 | (-0·01, 0·52) |  | 0·29 | (0·02, 0·56) |  | 39·1 (2·3) | -0·4 | (-0·9, 0·1) |  | -0·4 | (-0·9, 0·1) |  |
| 3 (1·751-2·126) | 3186 (526) | 57 | (-47, 161) |  | 48 | (-52, 147) |  | -0·12 (1·36) | 0·13 | (-0·15, 0·41) |  | 0·10 | (-0·18, 0·37) |  | 39·5 (2·4) | 0·0 | (-0·5, 0·5) |  | 0·0 | (-0·5, 0·5) |  |
| 4 (2·128-3·336) | 3116 (494) | -13 | (-113, 87) |  | -29 | (-125, 67) |  | -0·15 (1·25) | 0·09 | (-0·17, 0·36) |  | 0·06 | (-0·21, 0·33) |  | 39·2 (2·5) | -0·3 | (-0·9, 0·2) |  | -0·3 | (-0·9, 0·2) |  |
|  |  |  |  |  |  |  |  |  |  |  |  |  |  |  |  |  |  |  |  |  |  |
| Flagellin IgA (OD) |  |  |  | 0·06 |  |  | 0·07 |  |  |  | 0·32 |  |  | 0·44 |  |  |  | 0·15 |  |  | 0·13 |
| 1 (0·040-1·114) | 3218 (464) | Ref |  |  | Ref |  |  | -0·09 (1·28) | Ref |  |  | Ref |  |  | 39·7 (2·4) | Ref |  |  | Ref |  |  |
| 2 (1·121-1·604) | 3137 (513) | -81 | (-184, 23) |  | -65 | (-168, 37) |  | -0·09 (1·27) | 0·00 | (-0·27, 0·27) |  | 0·00 | (-0·28, 0·27) |  | 39·2 (2·4) | -0·5 | (-1·0, 0·0) |  | -0·5 | (-1·0, 0·1) |  |
| 3 (1·607-2·092) | 3123 (485) | -95 | (-196, 5) |  | -84 | (-185, 17) |  | -0·11 (1·29) | -0·02 | (-0·29, 0·25) |  | 0·01 | (-0·27, 0·29) |  | 39·1 (2·4) | -0·7 | (-1·2, -0·2) |  | -0·7 | (-1·2,  -0·1) |  |
| 4 (2·097-3·259) | 3117 (475) | -101 | (-202, -0) |  | -93 | (-193, 7) |  | -0·22 (1·26) | -0·13 | (-0·40, 0·14) |  | -0·11 | (-0·39, 0·17) |  | 39·4 (2·3) | -0·4 | (-0·9, 0·1) |  | -0·4 | (-0·9, 0·1) |  |
|  |  |  |  |  |  |  |  |  |  |  |  |  |  |  |  |  |  |  |  |  |  |
| LPS IgG (OD) |  |  |  | 0·96 |  |  | 0·71 |  |  |  | 0·88 |  |  | 0·96 |  |  |  | 0·75 |  |  | 0·63 |
| 1 (0·089-1·328) | 3138 (494) | Ref |  |  | Ref |  |  | -0·19 (1·34) | Ref |  |  | Ref |  |  | 39·5 (2·5) | Ref |  |  | Ref |  |  |
| 2 (1·329-1·802) | 3164 (464) | 27 | (-76, 129) |  | 19 | (-82, 121) |  | -0·04 (1·19) | 0·15 | (-0·12, 0·42) |  | 0·11 | (-0·16, 0·38) |  | 39·2 (2·2) | -0·3 | (-0·8, 0·2) |  | -0·2 | (-0·8, 0·3) |  |
| 3 (1·803-2·342) | 3149 (487) | 11 | (-93, 115) |  | 16 | (-84, 116) |  | -0·14 (1·23) | 0·05 | (-0·22, 0·33) |  | 0·05 | (-0·22, 0·32) |  | 39·4 (2·3) | -0·1 | (-0·6, 0·4) |  | -0·1 | (-0·6, 0·4) |  |
| 4 (2·346-3·367) | 3146 (499) | 8 | (-97, 114) |  | -18 | (-119, 83) |  | -0·13 (1·34) | 0·06 | (-0·23, 0·34) |  | 0·01 | (-0·27, 0·30) |  | 39·3 (2·5) | -0·2 | (-0·7, 0·4) |  | -0·2 | (-0·7, 0·3) |  |
|  |  |  |  |  |  |  |  |  |  |  |  |  |  |  |  |  |  |  |  |  |  |
| LPS IgA(OD) |  |  |  | 0·19 |  |  | 0·27 |  |  |  | 0·60 |  |  | 0·74 |  |  |  | 0·11 |  |  | 0·14 |
| 1 (0·017-1·005) | 3162 (479) | Ref |  |  | Ref |  |  | -0·22 (1·29) | Ref |  |  | Ref |  |  | 39·7 (2·3) | Ref |  |  | Ref |  |  |
| 2 (1·010-1·465) | 3156 (503) | -6 | (-110, 98) |  | -5 | (-107, 98) |  | 0·00 (1·28) | 0·22 | (-0·05, 0·49) |  | 0·22 | (-0·06, 0·49) |  | 39·1 (2·7) | -0·6 | (-1·1, -0·1) |  | -0·6 | (-1·1,  0·0) |  |
| 3 (1·468-2·054) | 3189 (492) | 27 | (-76, 130) |  | 20 | (-76, 116) |  | -0·02 (1·24) | 0·21 | (-0·06, 0·48) |  | 0·18 | (-0·09, 0·44) |  | 39·2 (2·3) | -0·5 | (-1·0, 0·0) |  | -0·4 | (-0·9, 0·1) |  |
| 4 (2·054-3·441) | 3088 (465) | -74 | (-175, 26) |  | -59 | (-156, 38) |  | -0·26 (1·26) | -0·04 | (-0·31, 0·23) |  | -0·01 | (-0·28, 0·26) |  | 39·3 (2·1) | -0·5 | (-0·9, 0·0) |  | -0·5 | (-0·9, 0·0) |  |
|  |  |  |  |  |  |  |  |  |  |  |  |  |  |  |  |  |  |  |  |  |  |
| sCD14 (ng/mL) |  |  |  | 0·76 |  |  | 0·52 |  |  |  | 0·59 |  |  | 0·50 |  |  |  | 0·62 |  |  | 0·72 |
| 1 (16·770-2362·485) | 3150 (489) | Ref |  |  | Ref |  |  | -0·10 (1·24) | Ref |  |  | Ref |  |  | 39·3 (2·4) | Ref |  |  | Ref |  |  |
| 2 (2365·170-3270·630) | 3133 (455) | -17 | (-117, 83) |  | -35 | (-135, 65) |  | -0·21 (1·27) | -0·11 | (-0·38, 0·15) |  | -0·14 | (-0·41, 0·13) |  | 39·4 (2·3) | 0·1 | (-0·4, 0·6) |  | 0·1 | (-0·4, 0·6) |  |
| 3 (3271·670-5002·015) | 3157 (492) | 7 | (-97, 112) |  | -0 | (-104, 103) |  | -0·12 (1·28) | -0·02 | (-0·29, 0·25) |  | -0·03 | (-0·30, 0·23) |  | 39·4 (2·5) | 0·1 | (-0·4, 0·6) |  | 0·1 | (-0·4, 0·6) |  |
| 4 (5005·980-19607·785) | 3158 (507) | 8 | (-97, 114) |  | 20 | (-82, 122) |  | -0·07 (1·31) | 0·03 | (-0·24, 0·30) |  | 0·04 | (-0·22, 0·31) |  | 39·2 (2·4) | -0·1 | (-0·6, 0·4) |  | 0·0 | (-0·5, 0·4) |  |
|  |  |  |  |  |  |  |  |  |  |  |  |  |  |  |  |  |  |  |  |  |  |
| I-FABP (pg/mL) |  |  |  | 0·89 |  |  | 0·96 |  |  |  | 0·96 |  |  | 0·79 |  |  |  | 0·59 |  |  | 0·58 |
| 1 (1·385-737·365) | 3145 (447) | Ref |  |  | Ref |  |  | -0·15 (1·31) | Ref |  |  | Ref |  |  | 39·4 (2·2) | Ref |  |  | Ref |  |  |
| 2 (739·435-1346·800) | 3187 (488) | 42 | (-57, 141) |  | 60 | (-38, 159) |  | -0·06 (1·21) | 0·09 | (-0·17, 0·36) |  | 0·14 | (-0·13, 0·41) |  | 39·5 (2·5) | 0·1 | (-0·4, 0·6) |  | 0·1 | (-0·4, 0·6) |  |
| 3 (1349·415- 2280·515) | 3111 (494) | -34 | (-134, 66) |  | -9 | (-105, 87) |  | -0·18 (1·26) | -0·03 | (-0·30, 0·24) |  | 0·00 | (-0·26, 0·27) |  | 39·3 (2·3) | -0·1 | (-0·6, 0·4) |  | -0·1 | (-0·6, 0·4) |  |
| 4 (2280·515- 8338·955) | 3156 (512) | 11 | (-92, 114) |  | 25 | (-77, 126) |  | -0·11 (1·33) | 0·04 | (-0·24, 0·32) |  | 0·08 | (-0·19, 0·36) |  | 39·3 (2·4) | -0·1 | (-0·6, 0·4) |  | -0·1 | (-0·6, 0·4) |  |
|  |  |  |  |  |  |  |  |  |  |  |  |  |  |  |  |  |  |  |  |  |  |
| **Systemic inflammation** |  |  |  |  |  |  |  |  |  |  |  |  |  |  |  |  |  |  |  |  |  |
| AGP (g/L) |  |  |  | **0·005** |  |  | **0·01** |  |  |  | 0·06 |  |  | 0·09 |  |  |  | 0·23 |  |  | 0·23 |
| 1 (0·180-0·680) | 3233 (492) | Ref |  |  | Ref |  |  | 0·05 (1·32) | Ref |  |  | Ref |  |  | 39·5 (2·6) | Ref |  |  | Ref |  |  |
| 2 (0·685-0·920) | 3134 (474) | -99 | (-202, 3) |  | -122 | (-223, -21) |  | -0·17 (1·18) | -0·22 | (-0·48, 0·05) |  | -0·27 | (-0·54, 0·00) |  | 39·3 (2·3) | -0·2 | (-0·7, 0·3) |  | -0·1 | (-0·7, 0·4) |  |
| 3 (0·925-1·295) | 3160 (467) | -73 | (-174, 28) |  | -62 | (-161 ,37) |  | -0·13 (1·19) | -0·18 | (-0·45, 0·08) |  | -0·18 | (-0·44, 0·09) |  | 39·4 (2·2) | -0·1 | (-0·6, 0·4) |  | 0·0 | (-0·5, 0·4) |  |
| 4 (1·300-8·040) | 3065 (497) | -168 | (-274, -62) |  | -163 | (-269, -57) |  | -0·27 (1·39) | -0·32 | (-0·61, -0·03) |  | -0·31 | (-0·60, -0·01) |  | 39·2 (2·4) | -0·4 | (-0·9, 0·2) |  | -0·3 | (-0·9, 0·2) |  |
|  |  |  |  |  |  |  |  |  |  |  |  |  |  |  |  |  |  |  |  |  |  |
| CRP (mg/L) |  |  |  | 0·38 |  |  | 0·26 |  |  |  | 0·76 |  |  | 0·81 |  |  |  | 0·32 |  |  | 0·24 |
| 1 (0·245-5·115) | 3145 (504) | Ref |  |  | Ref |  |  | -0·17 (1·27) | Ref |  |  | Ref |  |  | 39·5 (2·5) | Ref |  |  | Ref |  |  |
| 2 (5·150-11·460) | 3196 (450) | 51 | (-50, 152) |  | 25 | (-74, 124) |  | -0·03 (1·24) | 0·14 | (-0·13, 0·41) |  | 0·10 | (-0·16, 0·37) |  | 39·4 (2·3) | -0·1 | (-0·6, 0·4) |  | -0·2 | (-0·7, 0·3) |  |
| 3 (11·480- 20·910) | 3133 (495) | -12 | (-118, 94) |  | -27 | (-129, 76) |  | -0·15 (1·28) | 0·02 | (-0·25, 0·29) |  | 0·00 | (-0·27, 0·27) |  | 39·3 (2·3) | -0·2 | (-0·7, 0·3) |  | -0·3 | (-0·7, 0·2) |  |
| 4 (20·915-115·020) | 3123 (490) | -22 | (-128, 84) |  | -42 | (-144, 61) |  | -0·16 (1·30) | 0·01 | (-0·26, 0·29) |  | 0·01 | (-0·26, 0·28) |  | 39·2 (2·5) | -0·3 | (-0·8, 0·2) |  | -0·4 | (-0·9, 0·2) |  |
|  |  |  |  |  |  |  |  |  |  |  |  |  |  |  |  |  |  |  |  |  |  |
| **Growth hormone axis** |  |  |  |  |  |  |  |  |  |  |  |  |  |  |  |  |  |  |  |  |  |
| IGF-1 (ng/mL) |  |  |  | **0·004** |  |  | **0·003** |  |  |  | **0·01** |  |  | **0·01** |  |  |  | 0·79 |  |  | 0·97 |
| 1 (0·375-95·535) | 3066 (503) | Ref |  |  | Ref |  |  | -0·34 (1·31) | Ref |  |  | Ref |  |  | 39·5 (2·6) | Ref |  |  | Ref |  |  |
| 2 (96·445-170·920) | 3140 (440) | 74 | (-27, 175) |  | 75 | (-23, 174) |  | -0·10 (1·17) | 0·25 | (-0·02, 0·51) |  | 0·25 | (-0·02, 0·51) |  | 39·2 (2·3) | -0·3 | (-0·8, 0·3) |  | -0·3 | (-0·8, 0·2) |  |
| 3 (171·065-253·650) | 3158 (462) | 92 | (-12, 196) |  | 93 | (-9, 195) |  | -0·15 (1·27) | 0·20 | (-0·08, 0·47) |  | 0·19 | (-0·09, 0·47) |  | 39·5 (2·3) | 0·0 | (-0·5, 0·5) |  | 0·0 | (-0·5, 0·5) |  |
| 4 (253·985-1144·363) | 3233 (520) | 167 | (59, 276) |  | 172 | (64, 281) |  | 0·08 (1·31) | 0·42 | (0·14, 0·70) |  | 0·42 | (0·14, 0·70) |  | 39·3 (2·3) | -0·2 | (-0·7, 0·4) |  | -0·1 | (-0·7, 0·4) |  |
|  |  |  |  |  |  |  |  |  |  |  |  |  |  |  |  |  |  |  |  |  |  |
| FGF21 (pg/mL) |  |  |  | 0·11 |  |  | 0·09 |  |  |  | 0·89 |  |  | 0·92 |  |  |  | **0·02** |  |  | **0·03** |
| 1 (0·465-245·185) | 3220 (503) | Ref |  |  | Ref |  |  | -0·08 (1·26) | Ref |  |  | Ref |  |  | 39·6 (2·2) | Ref |  |  | Ref |  |  |
| 2 (246·975-575·790) | 3150 (444) | -70 | (-171, 31) |  | -53 | (-151, 44) |  | -0·13 (1·15) | -0·05 | (-0·31, 0·21) |  | -0·03 | (-0·28, 0·23) |  | 39·4 (2·5) | -0·2 | (-0·7, 0·3) |  | -0·2 | (-0·7, 0·3) |  |
| 3 (576·350-1465·635) | 3114 (477) | -106 | (-210, -2) |  | -92 | (-194, 9) |  | -0·22 (1·27) | -0·15 | (-0·42, 0·12) |  | -0·12 | (-0·39, 0·15) |  | 39·3 (2·2) | -0·3 | (-0·8, 0·2) |  | -0·3 | (-0·8, 0·2) |  |
| 4 (1468·845-9228·637) | 3114 (510) | -106 | (-214, 2) |  | -101 | (-204, 3) |  | -0·08 (1·41) | 0·00 | (-0·28, 0·28) |  | 0·00 | (-0·28, 0·28) |  | 39·0 (2·6) | -0·7 | (-1·2, -0·2) |  | -0·6 | (-1·1, -0·1) |  |

‡Adjusted models adjusted for age, BMI, marital status, education, parity, SES, clinic site, WHO HIV stage, CD4 T-cell count, timing of ART initiation, infant sex, and regimen.

OD, optical density; IgA, immunoglobulin A; IgG, immunoglobulin G; LPS, lipopolysaccharide; sCD14, soluble CD14; I-FABP, intestinal fatty acid-binding protein; CRP, C-reactive protein; AGP, α1-acid glycoprotein; IGF-1, insulin-like growth factor 1; FGF21, fibroblast growth factor 21; ng, nanograms; pg, picograms; mg, milligrams; mL, milliliters; L, liters; EED, environmental enteric dysfunction

**Supplemental Table 6. Univariable and multivariable association of analyte concentrations (optical density or concentration in quartiles) at 32 weeks gestation with stillbirth, low birthweight (LBW), small-for-gestational age (SGA), and preterm birth, among women living with HIV in Dar es Salaam Tanzania.^‡^**

|  | Stillbirth | | | | | | | Low birth weight | | | | | | | Small-for-gestational age | | | | | | | Preterm birth | | | | | | |
| --- | --- | --- | --- | --- | --- | --- | --- | --- | --- | --- | --- | --- | --- | --- | --- | --- | --- | --- | --- | --- | --- | --- | --- | --- | --- | --- | --- | --- |
|  |  | Unadjusted | | | Adjusted | | |  | Unadjusted | | | Adjusted | | |  | Unadjusted | | | Adjusted | | |  | Unadjusted | | | Adjusted | | |
|  | Events, n | RR | 95% CI | p | RR | 95% CI | p | Events, n | RR | 95% CI | p | RR | 95% CI | p | Events, n | RR | 95% CI | p | RR | 95% CI | p | Events, n | RR | 95% CI | p | RR | 95% CI | p |
| **Biomarkers of EED** |  |  |  |  |  |  |  |  |  |  |  |  |  |  |  |  |  |  |  |  |  |  |  |  |  |  |  |  |
| Flagellin IgG (OD) |  |  |  | 0·46 |  |  | 0·55 |  |  |  | 0·82 |  |  | 0·86 |  |  |  | 0·58 |  |  | 0·48 |  |  |  | 0·43 |  |  | 0·58 |
| 1 (0·087-1·385) | 5/177 |  |  |  | Ref |  |  | 11/172 |  |  |  | Ref |  |  | 35/172 |  |  |  | Ref |  |  | 23/172 |  |  |  | Ref |  |  |
| 2 (1·387-1·748) | 10/176 | 2·01 | (0·70,5·77) |  | 1·91 | (0·69, 5·30) |  | 8/166 | 0·75 | (0·31,1·83) |  | 0·55 | (0·21, 1·42) |  | 22/166 | 0·65 | (0·40,1·06) |  | 0·59 | (0·35, 0·99) |  | 24/166 | 1·08 | (0·64,1·84) |  | 1·06 | (0·62, 1·83) |  |
| 3 (1·751-2·126) | 9/176 | 1·81 | (0·62,5·30) |  | 1·74 | (0·62, 4·83) |  | 11/167 | 1·03 | (0·46,2·31) |  | 0·86 | (0·36, 2·05) |  | 35/167 | 1·03 | (0·68,1·56) |  | 1·04 | (0·68, 1·58) |  | 20/167 | 0·90 | (0·51,1·57) |  | 0·86 | (0·49, 1·52) |  |
| 4 (2·128-3·336) | 8/176 | 1·61 | (0·54,4·83) |  | 1·51 | (0·48, 4·71) |  | 11/168 | 1·02 | (0·46,2·30) |  | 0·95 | (0·40, 2·25) |  | 35/168 | 1·02 | (0·67,1·55) |  | 1·04 | (0·68, 1·57) |  | 29/168 | 1·29 | (0·78,2·14) |  | 1·22 | (0·73, 2·03) |  |
|  |  |  |  |  |  |  |  |  |  |  |  |  |  |  |  |  |  |  |  |  |  |  |  |  |  |  |  |  |
| Flagellin IgA (OD) |  |  |  | 0·15 |  |  | 0·17 |  |  |  | 0·48 |  |  | 0·63 |  |  |  | 0·38 |  |  | 0·52 |  |  |  | 0·23 |  |  | 0·24 |
| 1 (0·040-1·114) | 7/177 |  |  |  | Ref |  |  | 5/170 |  |  |  | Ref |  |  | 30/170 |  |  |  | Ref |  |  | 18/170 |  |  |  | Ref |  |  |
| 2 (1·121-1·604) | 6/176 | 0·86 | (0·30,2·52) |  | 0·78 | (0·26, 2·31) |  | 14/170 | 2·80 | (1·03,7·61) |  | 2·79 | (0·91, 8·53) |  | 31/170 | 1·03 | (0·66,1·63) |  | 1·08 | (0·68, 1·71) |  | 25/170 | 1·39 | (0·79,2·45) |  | 1·36 | (0·75, 2·46) |  |
| 3 (1·607-2·092) | 6/176 | 0·86 | (0·30,2·52) |  | 0·84 | (0·29, 2·43) |  | 14/170 | 2·80 | (1·03,7·61) |  | 3·15 | (1·04, 9·52) |  | 31/170 | 1·03 | (0·66,1·63) |  | 1·05 | (0·66, 1·67) |  | 29/170 | 1·61 | (0·93,2·79) |  | 1·68 | (0·96, 2·95) |  |
| 4 (2·097-3·259) | 13/176 | 1·87 | (0·76,4·57) |  | 1·67 | (0·70, 3·98) |  | 8/163 | 1·67 | (0·56,5·00) |  | 1·56 | (0·47, 5·18) |  | 35/163 | 1·22 | (0·79,1·89) |  | 1·17 | (0·75, 1·81) |  | 24/163 | 1·39 | (0·78,2·47) |  | 1·37 | (0·75, 2·51) |  |
|  |  |  |  |  |  |  |  |  |  |  |  |  |  |  |  |  |  |  |  |  |  |  |  |  |  |  |  |  |
| LPS IgG (OD) |  |  |  | 0·61 |  |  | 0·26 |  |  |  | 0·53 |  |  | 0·55 |  |  |  | 0·88 |  |  | 0·71 |  |  |  | 0·21 |  |  | 0·27 |
| 1 (0·089-1·328) | 7/177 |  |  |  | Ref |  |  | 13/170 |  |  |  | Ref |  |  | 35/170 |  |  |  | Ref |  |  | 21/170 |  |  |  | Ref |  |  |
| 2 (1·329-1·802) | 12/176 | 1·72 | (0·69,4·28) |  | 1·30 | (0·53, 3·19) |  | 8/164 | 0·64 | (0·27,1·50) |  | 0·61 | (0·23, 1·60) |  | 27/164 | 0·80 | (0·51,1·26) |  | 0·83 | (0·52, 1·34) |  | 21/164 | 1·04 | (0·59,1·83) |  | 0·98 | (0·57, 1·71) |  |
| 3 (1·803-2·342) | 6/176 | 0·86 | (0·30,2·52) |  | 0·65 | (0·23, 1·86) |  | 11/170 | 0·85 | (0·39,1·84) |  | 0·75 | (0·33, 1·71) |  | 30/170 | 0·86 | (0·55,1·33) |  | 0·85 | (0·54, 1·33) |  | 26/170 | 1·24 | (0·73,2·11) |  | 1·19 | (0·69, 2·04) |  |
| 4 (2·346-3·367) | 7/176 | 1·01 | (0·36,2·81) |  | 0·71 | (0·24, 2·05) |  | 9/169 | 0·70 | (0·31,1·59) |  | 0·73 | (0·31, 1·72) |  | 35/169 | 1·01 | (0·66,1·53) |  | 1·08 | (0·71, 1·64) |  | 28/169 | 1·34 | (0·79,2·27) |  | 1·29 | (0·76, 2·21) |  |
|  |  |  |  |  |  |  |  |  |  |  |  |  |  |  |  |  |  |  |  |  |  |  |  |  |  |  |  |  |
| LPS IgA(OD) |  |  |  | 0·07 |  |  | 0·07 |  |  |  | 0·70 |  |  | 0·60 |  |  |  | 0·38 |  |  | 0·41 |  |  |  | 0·39 |  |  | 0·51 |
| 1 (0·017-1·005) | 4/177 |  |  |  | Ref |  |  | 9/173 |  |  |  | Ref |  |  | 35/173 |  |  |  | Ref |  |  | 18/173 |  |  |  | Ref |  |  |
| 2 (1·010-1·465) | 8/176 | 2·01 | (0·62,6·56) |  | 2·19 | (0·67, 7·19) |  | 13/168 | 1·49 | (0·65,3·39) |  | 1·64 | (0·64, 4·19) |  | 27/168 | 0·79 | (0·50,1·25) |  | 0·80 | (0·51, 1·26) |  | 31/168 | 1·77 | (1·03,3·05) |  | 1·76 | (1·02, 3·04) |  |
| 3 (1·468-2·054) | 9/176 | 2·26 | (0·71,7·22) |  | 2·16 | (0·69, 6·74) |  | 11/167 | 1·27 | (0·54,2·98) |  | 1·25 | (0·51, 3·06) |  | 26/167 | 0·77 | (0·49,1·22) |  | 0·77 | (0·48, 1·21) |  | 21/167 | 1·21 | (0·67,2·19) |  | 1·13 | (0·62, 2·05) |  |
| 4 (2·054-3·441) | 11/176 | 2·77 | (0·90,8·53) |  | 2·83 | (0·92, 8·71) |  | 8/165 | 0·93 | (0·37,2·36) |  | 0·90 | (0·33, 2·46) |  | 39/165 | 1·17 | (0·78,1·75) |  | 1·16 | (0·78, 1·71) |  | 26/165 | 1·51 | (0·86,2·66) |  | 1·46 | (0·83, 2·59) |  |
|  |  |  |  |  |  |  |  |  |  |  |  |  |  |  |  |  |  |  |  |  |  |  |  |  |  |  |  |  |
| sCD14 (ng/mL) |  |  |  | 0·38 |  |  | 0·68 |  |  |  | 0·37 |  |  | 0·29 |  |  |  | 0·50 |  |  | 0·58 |  |  |  | 0·71 |  |  | 0·91 |
| 1 (16·770-2362·485) | 5/177 |  |  |  | Ref |  |  | 14/172 |  |  |  | Ref |  |  | 29/172 |  |  |  | Ref |  |  | 24/172 |  |  |  | Ref |  |  |
| 2 (2365·170-3270·630) | 7/176 | 1·41 | (0·46,4·36) |  | 1·34 | (0·44, 4·09) |  | 10/169 | 0·73 | (0·33,1·59) |  | 0·87 | (0·37, 2·01) |  | 33/169 | 1·16 | (0·74,1·82) |  | 1·24 | (0·79, 1·96) |  | 20/169 | 0·85 | (0·49,1·48) |  | 0·83 | (0·48, 1·43) |  |
| 3 (3271·670-5002·015) | 12/177 | 2·40 | (0·86,6·68) |  | 2·06 | (0·75, 5·60) |  | 8/165 | 0·60 | (0·26,1·38) |  | 0·56 | (0·22, 1·44) |  | 31/165 | 1·11 | (0·70,1·76) |  | 1·23 | (0·77, 1·96) |  | 28/165 | 1·22 | (0·74,2·01) |  | 1·14 | (0·70, 1·88) |  |
| 4 (5005·980-19607·785) | 8/176 | 1·61 | (0·54,4·83) |  | 1·33 | (0·48, 3·70) |  | 9/168 | 0·66 | (0·29,1·48) |  | 0·63 | (0·26, 1·55) |  | 34/168 | 1·20 | (0·77,1·88) |  | 1·19 | (0·76, 1·87) |  | 24/168 | 1·02 | (0·61,1·73) |  | 0·92 | (0·55, 1·53) |  |
|  |  |  |  |  |  |  |  |  |  |  |  |  |  |  |  |  |  |  |  |  |  |  |  |  |  |  |  |  |
| I-FABP (pg/mL) |  |  |  | **0·02** |  |  | **0·02** |  |  |  | 0·70 |  |  | 0·74 |  |  |  | 0·82 |  |  | 0·68 |  |  |  | 0·45 |  |  | 0·64 |
| 1 (1·385-737·365) | 5/177 |  |  |  | Ref |  |  | 10/172 |  |  |  | Ref |  |  | 33/172 |  |  |  | Ref |  |  | 24/172 |  |  |  | Ref |  |  |
| 2 (739·435-1346·800) | 5/176 | 1·01 | (0·30,3·42) |  | 1·06 | (0·32, 3·55) |  | 8/171 | 0·80 | (0·33,1·99) |  | 0·76 | (0·29, 2·00) |  | 33/171 | 1·01 | (0·65,1·55) |  | 1·03 | (0·66, 1·59) |  | 21/171 | 0·88 | (0·51,1·52) |  | 0·89 | (0·51, 1·54) |  |
| 3 (1349·415- 2280·515) | 9/177 | 1·80 | (0·61,5·27) |  | 2·04 | (0·75, 5·56) |  | 13/168 | 1·33 | (0·60,2·95) |  | 1·34 | (0·55, 3·26) |  | 31/168 | 0·96 | (0·62,1·50) |  | 0·97 | (0·62, 1·51) |  | 25/168 | 1·07 | (0·63,1·79) |  | 1·03 | (0·61, 1·74) |  |
| 4 (2280·515- 8338·955) | 13/176 | 2·61 | (0·95,7·18) |  | 2·44 | (1·00, 5·97) |  | 10/163 | 1·06 | (0·45,2·47) |  | 1·04 | (0·39, 2·80) |  | 30/163 | 0·96 | (0·61,1·50) |  | 0·93 | (0·60, 1·45) |  | 26/163 | 1·14 | (0·68,1·91) |  | 1·07 | (0·65, 1·76) |  |
|  |  |  |  |  |  |  |  |  |  |  |  |  |  |  |  |  |  |  |  |  |  |  |  |  |  |  |  |  |
| **Systemic inflammation** |  |  |  |  |  |  |  |  |  |  |  |  |  |  |  |  |  |  |  |  |  |  |  |  |  |  |  |  |
| AGP (g/L) |  |  |  | **0·03** |  |  | 0·11 |  |  |  | 0·86 |  |  | 0·88 |  |  |  | **0·02** |  |  | **0·03** |  |  |  | 0·80 |  |  | 0·57 |
| 1 (0·180-0·680) | 7/180 |  |  |  | Ref |  |  | 9/173 |  |  |  | Ref |  |  | 26/173 |  |  |  | Ref |  |  | 26/173 |  |  |  | Ref |  |  |
| 2 (0·685-0·920) | 4/173 | 0·59 | (0·18,2·00) |  | 0·58 | (0·17, 2·00) |  | 12/169 | 1·36 | (0·59,3·16) |  | 1·22 | (0·47, 3·20) |  | 31/169 | 1·22 | (0·76,1·97) |  | 1·31 | (0·81, 2·12) |  | 23/169 | 0·91 | (0·54,1·52) |  | 0·84 | (0·50, 1·41) |  |
| 3 (0·925-1·295) | 7/178 | 1·01 | (0·36,2·83) |  | 0·88 | (0·34, 2·31) |  | 10/171 | 1·12 | (0·47,2·70) |  | 0·98 | (0·35, 2·78) |  | 29/171 | 1·13 | (0·69,1·83) |  | 1·17 | (0·72, 1·90) |  | 25/171 | 0·97 | (0·59,1·62) |  | 0·88 | (0·53, 1·46) |  |
| 4 (1·300-8·040) | 14/175 | 2·06 | (0·85,4·98) |  | 1·63 | (0·66, 4·00) |  | 10/161 | 1·19 | (0·50,2·86) |  | 1·02 | (0·37, 2·79) |  | 41/161 | 1·69 | (1·09,2·64) |  | 1·69 | (1·07, 2·65) |  | 22/161 | 0·91 | (0·54,1·54) |  | 0·83 | (0·49, 1·40) |  |
|  |  |  |  |  |  |  |  |  |  |  |  |  |  |  |  |  |  |  |  |  |  |  |  |  |  |  |  |  |
| CRP (mg/L) |  |  |  | 0·06 |  |  | 0·20 |  |  |  | 0·73 |  |  | 0·67 |  |  |  | 0·23 |  |  | 0·25 |  |  |  | 0·82 |  |  | 0·73 |
| 1 (0·245-5·115) | 4/177 |  |  |  | Ref |  |  | 13/173 |  |  |  | Ref |  |  | 35/173 |  |  |  | Ref |  |  | 25/173 |  |  |  | Ref |  |  |
| 2 (5·150-11·460) | 7/176 | 1·76 | (0·52,5·91) |  | 1·40 | (0·39, 5·01) |  | 7/169 | 0·55 | (0·23,1·35) |  | 0·61 | (0·23, 1·63) |  | 25/169 | 0·73 | (0·46,1·17) |  | 0·76 | (0·47, 1·22) |  | 26/169 | 1·06 | (0·64,1·77) |  | 1·15 | (0·70, 1·91) |  |
| 3 (11·480- 20·910) | 10/177 | 2·50 | (0·80,7·83) |  | 1·84 | (0·62, 5·40) |  | 12/167 | 0·96 | (0·45,2·04) |  | 0·94 | (0·42, 2·12) |  | 29/167 | 0·86 | (0·55,1·34) |  | 0·89 | (0·56, 1·40) |  | 19/167 | 0·79 | (0·45,1·38) |  | 0·84 | (0·48, 1·49) |  |
| 4 (20·915-115·020) | 11/176 | 2·77 | (0·90,8·53) |  | 2·03 | (0·67, 6·13) |  | 9/165 | 0·73 | (0·32,1·65) |  | 0·72 | (0·29, 1·80) |  | 38/165 | 1·14 | (0·76,1·71) |  | 1·14 | (0·75, 1·71) |  | 26/165 | 1·09 | (0·66,1·81) |  | 1·15 | (0·69, 1·90) |  |
|  |  |  |  |  |  |  |  |  |  |  |  |  |  |  |  |  |  |  |  |  |  |  |  |  |  |  |  |  |
| **Growth hormone axis** |  |  |  |  |  |  |  |  |  |  |  |  |  |  |  |  |  |  |  |  |  |  |  |  |  |  |  |  |
| IGF-1 (ng/mL) |  |  |  | 0·19 |  |  | 0·08 |  |  |  | 0·56 |  |  | 0·44 |  |  |  | 0·11 |  |  | 0·14 |  |  |  | 0·66 |  |  | 0·75 |
| 1 (0·375-95·535) | 10/177 |  |  |  | Ref |  |  | 12/167 |  |  |  | Ref |  |  | 40/167 |  |  |  | Ref |  |  | 23/167 |  |  |  | Ref |  |  |
| 2 (96·445-170·920) | 7/176 | 0·70 | (0·27,1·81) |  | 0·67 | (0·26, 1·69) |  | 11/169 | 0·91 | (0·41,2·00) |  | 0·85 | (0·36, 2·04) |  | 27/169 | 0·67 | (0·43,1·03) |  | 0·65 | (0·42, 1·02) |  | 26/169 | 1·12 | (0·66,1·88) |  | 1·14 | (0·68, 1·92) |  |
| 3 (171·065-253·650) | 11/177 | 1·10 | (0·48,2·53) |  | 1·06 | (0·47, 2·40) |  | 8/166 | 0·67 | (0·28,1·60) |  | 0·62 | (0·23, 1·67) |  | 34/166 | 0·86 | (0·57,1·28) |  | 0·88 | (0·58, 1·34) |  | 18/166 | 0·79 | (0·44,1·40) |  | 0·83 | (0·47, 1·45) |  |
| 4 (253·985-1144·363) | 4/176 | 0·40 | (0·13,1·26) |  | 0·30 | (0·08, 1·08) |  | 10/172 | 0·81 | (0·36,1·82) |  | 0·69 | (0·24, 1·97) |  | 26/172 | 0·63 | (0·40,0·99) |  | 0·64 | (0·40, 1·00) |  | 29/172 | 1·22 | (0·74,2·03) |  | 1·20 | (0·72, 2·00) |  |
|  |  |  |  |  |  |  |  |  |  |  |  |  |  |  |  |  |  |  |  |  |  |  |  |  |  |  |  |  |
| FGF21 (pg/mL) |  |  |  | 0·30 |  |  | 0·22 |  |  |  | 0·69 |  |  | 0·87 |  |  |  | 0·12 |  |  | 0·10 |  |  |  | **0·004** |  |  | **0·02** |
| 1 (0·465-245·185) | 5/177 |  |  |  | Ref |  |  | 9/172 |  |  |  | Ref |  |  | 28/172 |  |  |  | Ref |  |  | 19/172 |  |  |  | Ref |  |  |
| 2 (246·975-575·790) | 9/176 | 1·81 | (0·62,5·30) |  | 2·20 | (0·83, 5·87) |  | 11/167 | 1·26 | (0·54,2·96) |  | 1·22 | (0·51, 2·91) |  | 29/167 | 1·07 | (0·66,1·71) |  | 1·09 | (0·68, 1·73) |  | 19/167 | 1·03 | (0·57,1·88) |  | 1·04 | (0·57, 1·90) |  |
| 3 (576·350-1465·635) | 8/177 | 1·60 | (0·53,4·80) |  | 1·59 | (0·59, 4·29) |  | 10/169 | 1·13 | (0·47,2·71) |  | 1·02 | (0·39, 2·65) |  | 33/169 | 1·20 | (0·76,1·89) |  | 1·20 | (0·76, 1·89) |  | 23/169 | 1·23 | (0·70,2·18) |  | 1·20 | (0·68, 2·11) |  |
| 4 (1468·845-9228·637) | 10/176 | 2·01 | (0·70,5·77) |  | 2·26 | (0·89, 5·75) |  | 11/166 | 1·27 | (0·54,2·98) |  | 1·15 | (0·44, 2·97) |  | 37/166 | 1·37 | (0·88,2·13) |  | 1·42 | (0·91, 2·22) |  | 35/166 | 1·91 | (1·14,3·20) |  | 1·77 | (1·04, 3·00) |  |

‡Adjusted models adjusted for age, BMI, marital status, education, parity, SES, clinic site, WHO HIV stage, CD4 T-cell count, timing of ART initiation, infant sex, and regimen.

OD, optical density; IgA, immunoglobulin A; IgG, immunoglobulin G; LPS, lipopolysaccharide; sCD14, soluble CD14; I-FABP, intestinal fatty acid-binding protein; CRP, C-reactive protein; AGP, α1-acid glycoprotein; IGF-1, insulin-like growth factor 1; FGF21, fibroblast growth factor 21; ng, nanograms; pg, picograms; mg, milligrams; mL, milliliters; L, liters; EED, environmental enteric dysfunction

**Supplemental Table 7. Univariable and multivariable association of analyte concentrations (optical density or concentration in quartiles) at 32 weeks gestation with infant birthweight, birthweight-for-gestational age z-score, and gestational age at birth, among women living with HIV in Dar es Salaam Tanzania, additionally adjusted for C-reactive protein.^‡^**

|  | Birthweight | | | | | | | Birthweight-for-gestational age z score | | | | | | | Gestational age at birth | | | | | | |
| --- | --- | --- | --- | --- | --- | --- | --- | --- | --- | --- | --- | --- | --- | --- | --- | --- | --- | --- | --- | --- | --- |
|  |  | Unadjusted | | | Adjusted | | |  | Unadjusted | | | Adjusted | | |  | Unadjusted | | | Adjusted | | |
|  | Mean (SD) | Mean diff (g) | 95% CI | p | Mean diff (g) | 95% CI | p | Mean (SD) | Mean diff (z-score) | 95% CI | p | Mean diff (z-score) | 95% CI | p | Mean (SD) | Mean diff (wks) | 95% CI | p | Mean diff (wks) | 95% CI | p |
| **Biomarkers of EED** |  |  |  |  |  |  |  |  |  |  |  |  |  |  |  |  |  |  |  |  |  |
| Flagellin IgG (OD) |  |  |  | 0·90 |  |  | 0·61 |  |  |  | 0·67 |  |  | 0·90 |  |  |  | 0·39 |  |  | 0·46 |
| 1 (0·087-1·385) | 3129 (449) | Ref |  |  | Ref |  |  | -0·24 (1·27) | Ref |  |  | Ref |  |  | 39·5 (2·4) | Ref |  |  | Ref |  |  |
| 2 (1·387-1·748) | 3166 (471) | 37 | (-61, 135) |  | 66 | (-33, 164) |  | 0·01 (1·21) | 0·25 | (-0·01, 0·52) |  | 0·29 | (0·02, 0·56) |  | 39·1 (2·3) | -0·4 | (-0·9, 0·1) |  | -0·4 | (-0·9, 0·1) |  |
| 3 (1·751-2·126) | 3186 (526) | 57 | (-47, 161) |  | 55 | (-45, 155) |  | -0·12 (1·36) | 0·13 | (-0·15, 0·41) |  | 0·10 | (-0·18, 0·38) |  | 39·5 (2·4) | 0·0 | (-0·5, 0·5) |  | 0·0 | (-0·5, 0·5) |  |
| 4 (2·128-3·336) | 3116 (494) | -13 | (-113, 87) |  | -24 | (-121, 72) |  | -0·15 (1·25) | 0·09 | (-0·17, 0·36) |  | 0·06 | (-0·20, 0·33) |  | 39·2 (2·5) | -0·3 | (-0·9, 0·2) |  | -0·3 | (-0·8, 0·2) |  |
|  |  |  |  |  |  |  |  |  |  |  |  |  |  |  |  |  |  |  |  |  |  |
| Flagellin IgA (OD) |  |  |  | 0·06 |  |  | 0·07 |  |  |  | 0·32 |  |  | 0·42 |  |  |  | 0·15 |  |  | 0·13 |
| 1 (0·040-1·114) | 3218 (464) | Ref |  |  | Ref |  |  | -0·09 (1·28) | Ref |  |  | Ref |  |  | 39·7 (2·4) | Ref |  |  | Ref |  |  |
| 2 (1·121-1·604) | 3137 (513) | -81 | (-184, 23) |  | -66 | (-169, 36) |  | -0·09 (1·27) | 0·00 | (-0·27, 0·27) |  | 0·00 | (-0·28, 0·27) |  | 39·2 (2·4) | -0·5 | (-1·0, -0·0) |  | -0·5 | (-1·0, 0·1) |  |
| 3 (1·607-2·092) | 3123 (485) | -95 | (-196, 5) |  | -83 | (-185, 18) |  | -0·11 (1·29) | -0·02 | (-0·29, 0·25) |  | 0·01 | (-0·28, 0·29) |  | 39·1 (2·4) | -0·7 | (-1·2, -0·2) |  | -0·7 | (-1·2, -0·1) |  |
| 4 (2·097-3·259) | 3117 (475) | -101 | (-202, -0) |  | -96 | (-196, 4) |  | -0·22 (1·26) | -0·13 | (-0·40, 0·14) |  | -0·12 | (-0·39, 0·16) |  | 39·4 (2·3) | -0·4 | (-0·9, 0·1) |  | -0·4 | (-0·9, 0·1) |  |
|  |  |  |  |  |  |  |  |  |  |  |  |  |  |  |  |  |  |  |  |  |  |
| LPS IgG (OD) |  |  |  | 0·96 |  |  | 0·72 |  |  |  | 0·88 |  |  | 0·96 |  |  |  | 0·75 |  |  | 0·64 |
| 1 (0·089-1·328) | 3138 (494) | Ref |  |  | Ref |  |  | -0·19 (1·34) | Ref |  |  | Ref |  |  | 39·5 (2·5) | Ref |  |  | Ref |  |  |
| 2 (1·329-1·802) | 3164 (464) | 27 | (-76, 129) |  | 20 | (-81, 122) |  | -0·04 (1·19) | 0·15 | (-0·12, 0·42) |  | 0·12 | (-0·16, 0·39) |  | 39·2 (2·2) | -0·3 | (-0·8, 0·2) |  | -0·3 | (-0·8, 0·2) |  |
| 3 (1·803-2·342) | 3149 (487) | 11 | (-93, 115) |  | 18 | (-82, 118) |  | -0·14 (1·23) | 0·05 | (-0·22, 0·33) |  | 0·05 | (-0·22, 0·32) |  | 39·4 (2·3) | -0·1 | (-0·6, 0·4) |  | -0·1 | (-0·6, 0·4) |  |
| 4 (2·346-3·367) | 3146 (499) | 8 | (-97, 114) |  | -17 | (-119, 84) |  | -0·13 (1·34) | 0·06 | (-0·23, 0·34) |  | 0·02 | (-0·27, 0·30) |  | 39·3 (2·5) | -0·2 | (-0·7, 0·4) |  | -0·2 | (-0·7, 0·3) |  |
|  |  |  |  |  |  |  |  |  |  |  |  |  |  |  |  |  |  |  |  |  |  |
| LPS IgA (OD) |  |  |  | 0·19 |  |  | 0·27 |  |  |  | 0·60 |  |  | 0·73 |  |  |  | 0·11 |  |  | 0·15 |
| 1 (0·017-1·005) | 3162 (479) | Ref |  |  | Ref |  |  | -0·22 (1·29) | Ref |  |  | Ref |  |  | 39·7 (2·3) | Ref |  |  | Ref |  |  |
| 2 (1·010-1·465) | 3156 (503) | -6 | (-110, 98) |  | -3 | (-105, 100) |  | 0·00 (1·28) | 0·22 | (-0·05, 0·49) |  | 0·22 | (-0·05, 0·50) |  | 39·1 (2·7) | -0·6 | (-1·1, -0·1) |  | -0·6 | (-1·1, 0·0) |  |
| 3 (1·468-2·054) | 3189 (492) | 27 | (-76, 130) |  | 19 | (-77, 114) |  | -0·02 (1·24) | 0·21 | (-0·06, 0·48) |  | 0·18 | (-0·08, 0·44) |  | 39·2 (2·3) | -0·5 | (-1·0, 0·0) |  | -0·4 | (-0·9, 0·1) |  |
| 4 (2·054-3·441) | 3088 (465) | -74 | (-175, 26) |  | -58 | (-155, 39) |  | -0·26 (1·26) | -0·04 | (-0·31, 0·23) |  | -0·01 | (-0·28, 0·26) |  | 39·3 (2·1) | -0·5 | (-0·9, 0·0) |  | -0·5 | (-0·9, 0·0) |  |
|  |  |  |  |  |  |  |  |  |  |  |  |  |  |  |  |  |  |  |  |  |  |
| sCD14 (ng/mL) |  |  |  | 0·76 |  |  | 0·29 |  |  |  | 0·59 |  |  | 0·44 |  |  |  | 0·62 |  |  | 0·92 |
| 1 (16·770-2362·485) | 3150 (489) | Ref |  |  | Ref |  |  | -0·10 (1·24) | Ref |  |  | Ref |  |  | 39·3 (2·4) | Ref |  |  | Ref |  |  |
| 2 (2365·170-3270.630) | 3133 (455) | -17 | (-117, 83) |  | -33 | (-134, 68) |  | -0·21 (1·27) | -0·11 | (-0·38, 0·15) |  | -0·14 | (-0·41, 0·13) |  | 39·4 (2·3) | 0·1 | (-0·4, 0·6) |  | 0·1 | (-0·3, 0·6) |  |
| 3 (3271.670-5002.015) | 3157 (492) | 7 | (-97, 112) |  | 6 | (-102, 114) |  | -0·12 (1·28) | -0·02 | (-0·29, 0·25) |  | -0·03 | (-0·31, 0·24) |  | 39·4 (2·5) | 0·1 | (-0·4, 0·6) |  | 0·2 | (-0·3, 0·7) |  |
| 4 (5005.980-19607·785) | 3158 (507) | 8 | (-97, 114) |  | 44 | (-64, 151) |  | -0·07 (1·31) | 0·03 | (-0·24, 0·30) |  | 0·07 | (-0·22, 0·35) |  | 39·2 (2·4) | -0·1 | (-0·6, 0·4) |  | 0·1 | (-0·5, 0·6) |  |
|  |  |  |  |  |  |  |  |  |  |  |  |  |  |  |  |  |  |  |  |  |  |
| I-FABP (pg/mL) |  |  |  | 0·89 |  |  | 0·80 |  |  |  | 0·96 |  |  | 0·73 |  |  |  | 0·59 |  |  | 0·70 |
| 1 (1·385-737.365) | 3145 (447) | Ref |  |  | Ref |  |  | -0·15 (1·31) | Ref |  |  | Ref |  |  | 39·4 (2·2) | Ref |  |  | Ref |  |  |
| 2 (739.435-1346.800) | 3187 (488) | 42 | (-57, 141) |  | 67 | (-31, 165) |  | -0·06 (1·21) | 0·09 | (-0·17, 0·36) |  | 0·15 | (-0·12, 0·41) |  | 39·5 (2·5) | 0·1 | (-0·4, 0·6) |  | 0·1 | (-0·4, 0·7) |  |
| 3 (1349.415- 2280.515) | 3111 (494) | -34 | (-134, 66) |  | -4 | (-100, 92) |  | -0·18 (1·26) | -0·03 | (-0·30, 0·24) |  | 0·01 | (-0·26, 0·27) |  | 39·3 (2·3) | -0·1 | (-0·6, 0·4) |  | 0·0 | (-0·5, 0·5) |  |
| 4 (2280.515- 8338·955) | 3156 (512) | 11 | (-92, 114) |  | 37 | (-64, 138) |  | -0·11 (1·33) | 0·04 | (-0·24,0·32) |  | 0·10 | (-0·18, 0·37) |  | 39·3 (2·4) | -0·1 | (-0·6, 0·4) |  | 0·0 | (-0·5, 0·5) |  |
|  |  |  |  |  |  |  |  |  |  |  |  |  |  |  |  |  |  |  |  |  |  |
| **Systemic inflammation** |  |  |  |  |  |  |  |  |  |  |  |  |  |  |  |  |  |  |  |  |  |
| AGP (g/L) |  |  |  | **0·005** |  |  | **0·02** |  |  |  | 0·06 |  |  | 0·08 |  |  |  | 0·23 |  |  | 0·45 |
| 1 (0·180-0·680) | 3233 (492) | Ref |  |  | Ref |  |  | 0·05 (1·32) | Ref |  |  | Ref |  |  | 39·5 (2·6) | Ref |  |  | Ref |  |  |
| 2 (0·685-0·920) | 3134 (474) | -99 | (-202, 3) |  | -127 | (-233, -20) |  | -0·17 (1·18) | -0·22 | (-0·48, 0·05) |  | -0·30 | (-0·58, -0·02) |  | 39·3 (2·3) | -0·2 | (-0·7, 0·3) |  | -0·1 | (-0·6, 0·4) |  |
| 3 (0·925-1·295) | 3160 (467) | -73 | (-174, 28) |  | -63 | (-170, 44) |  | -0·13 (1·19) | -0·18 | (-0·45 ,0·08) |  | -0·21 | (-0·50, 0·07) |  | 39·4 (2·2) | -0·1 | (-0·6, 0·4) |  | 0·0 | (-0·5, 0·6) |  |
| 4 (1·300-8·040) | 3065 (497) | -168 | (-274, -62) |  | -163 | (-279, -48) |  | -0·27 (1·39) | -0·32 | (-0·61, -0·03) |  | -0·35 | (-0·67, -0·03) |  | 39·2 (2·4) | -0·4 | (-0·9, 0·2) |  | -0·2 | (-0·8, 0·3) |  |
|  |  |  |  |  |  |  |  |  |  |  |  |  |  |  |  |  |  |  |  |  |  |
| **Growth hormone axis** |  |  |  |  |  |  |  |  |  |  |  |  |  |  |  |  |  |  |  |  |  |
| IGF-1 (ng/mL) |  |  |  | **0·004** |  |  | **0·002** |  |  |  | **0·009** |  |  | **0·008** |  |  |  | 0·79 |  |  | 0·98 |
| 1 (0·375-95·535) | 3066 (503) | Ref |  |  | Ref |  |  | -0·34 (1·31) | Ref |  |  | Ref |  |  | 39·5 (2·6) | Ref |  |  | Ref |  |  |
| 2 (96.445-170.920) | 3140 (440) | 74 | (-27, 175) |  | 82 | (-16, 180) |  | -0·10 (1·17) | 0·25 | (-0·02, 0·51) |  | 0·25 | (-0·01, 0·52) |  | 39·2 (2·3) | -0·3 | (-0·8, 0·3) |  | -0·3 | (-0·8, 0·3) |  |
| 3 (171·065-253.650) | 3158 (462) | 92 | (-12, 196) |  | 99 | (-3, 200) |  | -0·15 (1·27) | 0·20 | (-0·08, 0·47) |  | 0·20 | (-0·08, 0·47) |  | 39·5 (2·3) | 0·0 | (-0·5, 0·5) |  | 0·0 | (-0·5, 0·5) |  |
| 4 (253.985-1144.363) | 3233 (520) | 167 | (59, 276) |  | 176 | (69, 284) |  | 0·08 (1·31) | 0·42 | (0·14, 0·70) |  | 0·43 | (0·15, 0·70) |  | 39·3 (2·3) | -0·2 | (-0·7, 0·4) |  | -0·1 | (-0·6, 0·4) |  |
|  |  |  |  |  |  |  |  |  |  |  |  |  |  |  |  |  |  |  |  |  |  |
| FGF21 (pg/mL) |  |  |  | 0·11 |  |  | 0·14 |  |  |  | 0·89 |  |  | 0·82 |  |  |  | **0·02** |  |  | **0·03** |
| 1 (0·465-245·185) | 3220 (503) | Ref |  |  | Ref |  |  | -0·08 (1·26) | Ref |  |  | Ref |  |  | 39·6 (2·2) | Ref |  |  | Ref |  |  |
| 2 (246·975-575·790) | 3150 (444) | -70 | (-171, 31) |  | -50 | (-149, 48) |  | -0·13 (1·15) | -0·05 | (-0·31, 0·21) |  | -0·02 | (-0·28, 0·23) |  | 39·4 (2·5) | -0·2 | (-0·7, 0·3) |  | -0·2 | (-0·6, 0·3) |  |
| 3 (576·350-1465·635) | 3114 (477) | -106 | (-210, -2) |  | -89 | (-191, 14) |  | -0·22 (1·27) | -0·15 | (-0·42, 0·12) |  | -0·12 | (-0·39, 0·15) |  | 39·3 (2·2) | -0·3 | (-0·8, 0·2) |  | -0·3 | (-0·7, 0·2) |  |
| 4 (1468·845-9228.637) | 3114 (510) | -106 | (-214, 2) |  | -91 | (-196, 14) |  | -0·08 (1·41) | 0·00 | (-0·28, 0·28) |  | 0·02 | (-0·26, 0·30) |  | 39·0 (2·6) | -0·7 | (-1·2, -0·2) |  | -0·6 | (-1·1, -0·1) |  |

‡Adjusted models adjusted for C-reactive protein, age, BMI, marital status, education, parity, SES, clinic site, WHO HIV stage, CD4 T-cell count, timing of ART initiation, infant sex, and regimen.

OD, optical density; IgA, immunoglobulin A; IgG, immunoglobulin G; LPS, lipopolysaccharide; sCD14, soluble CD14; I-FABP, intestinal fatty acid-binding protein; CRP, C-reactive protein; AGP, α1-acid glycoprotein; IGF-1, insulin-like growth factor 1; FGF21, fibroblast growth factor 21; ng, nanograms; pg, picograms; mg, milligrams; mL, milliliters; L, liters; EED, environmental enteric dysfunction

**Supplemental Table 8. Univariable and multivariable association of analyte concentrations (optical density or concentration in quartiles) at 32 weeks gestation with low birthweight (LBW), small-for-gestational age (SGA), and preterm birth, among women living with HIV in Dar es Salaam Tanzania, additionally adjusted for C-reactive protein.^‡^**

|  | Stillbirth | | | | | | | Low birth weight | | | | | | | Small-for-gestational age | | | | | | | Preterm birth | | | | | | |
| --- | --- | --- | --- | --- | --- | --- | --- | --- | --- | --- | --- | --- | --- | --- | --- | --- | --- | --- | --- | --- | --- | --- | --- | --- | --- | --- | --- | --- |
|  |  | Unadjusted | | | Adjusted | | |  | Unadjusted | | | Adjusted | | |  | Unadjusted | | | Adjusted | | |  | Unadjusted | | | Adjusted | | |
|  | Events, n | RR | 95% CI | p | RR | 95% CI | p | Events, n | RR | 95% CI | p | RR | 95% CI | p | Events, n | RR | 95% CI | p | RR | 95% CI | p | Events, n | RR | 95% CI | p | RR | 95% CI | p |
| **Biomarkers of EED** |  |  |  |  |  |  |  |  |  |  |  |  |  |  |  |  |  |  |  |  |  |  |  |  |  |  |  |  |
| Flagellin IgG (OD) |  |  |  | 0·46 |  |  | 0·62 |  |  |  | 0·82 |  |  | 0·78 |  |  |  | 0·58 |  |  | 0·54 |  |  |  | 0·43 |  |  | 0·57 |
| 1 (0·087-1·385) | 5/177 | Ref |  |  | Ref |  |  | 11/172 | Ref |  |  | Ref |  |  | 35/172 | Ref |  |  | Ref |  |  | 23/172 | Ref |  |  | Ref |  |  |
| 2 (1·387-1·748) | 10/176 | 2·01 | (0·70, 5·77) |  | 1·89 | (0·68, 5·24) |  | 8/166 | 0·75 | (0·31, 1·83) |  | 0·60 | (0·25, 1·45) |  | 22/166 | 0·65 | (0·40, 1·06) |  | 0·60 | (0·36, 1·00) |  | 24/166 | 1·08 | (0·64, 1·84) |  | 1·06 | (0·62, 1·83) |  |
| 3 (1·751-2·126) | 9/176 | 1·81 | (0·62, 5·30) |  | 1·66 | (0·58, 4·78) |  | 11/167 | 1·03 | (0·46, 2·31) |  | 0·95 | (0·42, 2·17) |  | 35/167 | 1·03 | (0·68, 1·56) |  | 1·02 | (0·67, 1·57) |  | 20/167 | 0·90 | (0·51, 1·57) |  | 0·87 | (0·49, 1·53) |  |
| 4 (2·128-3·336) | 8/176 | 1·61 | (0·54, 4·83) |  | 1·44 | (0·47, 4·43) |  | 11/168 | 1·02 | (0·46, 2·30) |  | 1·01 | (0·45, 2·27) |  | 35/168 | 1·02 | (0·67, 1·55) |  | 1·03 | (0·67, 1·57) |  | 29/168 | 1·29 | (0·78, 2·14) |  | 1·22 | (0·73, 2·04) |  |
|  |  |  |  |  |  |  |  |  |  |  |  |  |  |  |  |  |  |  |  |  |  |  |  |  |  |  |  |  |
| Flagellin IgA (OD) |  |  |  | 0·15 |  |  | 0·16 |  |  |  | 0·48 |  |  | 0·56 |  |  |  | 0·38 |  |  | 0·44 |  |  |  | 0·23 |  |  | 0·23 |
| 1 (0·040-1·114) | 7/177 | Ref |  |  | Ref |  |  | 5/170 | Ref |  |  | Ref |  |  | 30/170 | Ref |  |  | Ref |  |  | 18/170 | Ref |  |  | Ref |  |  |
| 2 (1·121-1·604) | 6/176 | 0·86 | (0·30, 2·52) |  | 0·81 | (0·26, 2·49) |  | 14/170 | 2·80 | (1·03, 7·61) |  | 2·58 | (0·90, 7·43) |  | 31/170 | 1·03 | (0·66, 1·63) |  | 1·09 | (0·69, 1·72) |  | 25/170 | 1·39 | (0·79, 2·45) |  | 1·39 | (0·76, 2·54) |  |
| 3 (1·607-2·092) | 6/176 | 0·86 | (0·30, 2·52) |  | 0·84 | (0·29, 2·46) |  | 14/170 | 2·80 | (1·03, 7·61) |  | 2·92 | (1·06, 8·04) |  | 31/170 | 1·03 | (0·66, 1·63) |  | 1·07 | (0·67, 1·69) |  | 29/170 | 1·61 | (0·93, 2·79) |  | 1·70 | (0·96, 2·98) |  |
| 4 (2·097-3·259) | 13/176 | 1·87 | (0·76, 4·57) |  | 1·75 | (0·70, 4·34) |  | 8/163 | 1·67 | (0·56, 5·00) |  | 1·57 | (0·50, 4·89) |  | 35/163 | 1·22 | (0·79, 1·89) |  | 1·20 | (0·77, 1·86) |  | 24/163 | 1·39 | (0·78, 2·47) |  | 1·40 | (0·76, 2·57) |  |
|  |  |  |  |  |  |  |  |  |  |  |  |  |  |  |  |  |  |  |  |  |  |  |  |  |  |  |  |  |
| LPS IgG (OD) |  |  |  | 0·61 |  |  | 0·24 |  |  |  | 0·53 |  |  | 0·58 |  |  |  | 0·88 |  |  | 0·71 |  |  |  | 0·21 |  |  | 0·27 |
| 1 (0·089-1·328) | 7/177 | Ref |  |  | Ref |  |  | 13/170 | Ref |  |  | Ref |  |  | 35/170 | Ref |  |  | Ref |  |  | 21/170 | Ref |  |  | Ref |  |  |
| 2 (1·329-1·802) | 12/176 | 1·72 | (0·69, 4·28) |  | 1·30 | (0·53, 3·17) |  | 8/164 | 0·64 | (0·27, 1·50) |  | 0·64 | (0·26, 1·60) |  | 27/164 | 0·80 | (0·51, 1·26) |  | 0·82 | (0·51, 1·32) |  | 21/164 | 1·04 | (0·59, 1·83) |  | 0·97 | (0·56, 1·69) |  |
| 3 (1·803-2·342) | 6/176 | 0·86 | (0·30, 2·52) |  | 0·61 | (0·21, 1·79) |  | 11/170 | 0·85 | (0·39, 1·84) |  | 0·81 | (0·36, 1·82) |  | 30/170 | 0·86 | (0·55, 1·33) |  | 0·84 | (0·53, 1·33) |  | 26/170 | 1·24 | (0·73, 2·11) |  | 1·17 | (0·68, 2·00) |  |
| 4 (2·346-3·367) | 7/176 | 1·01 | (0·36, 2·81) |  | 0·71 | (0·25, 2·05) |  | 9/169 | 0·70 | (0·31, 1·59) |  | 0·74 | (0·33, 1·68) |  | 35/169 | 1·01 | (0·66, 1·53) |  | 1·07 | (0·70, 1·64) |  | 28/169 | 1·34 | (0·79, 2·27) |  | 1·29 | (0·76, 2·20) |  |
|  |  |  |  |  |  |  |  |  |  |  |  |  |  |  |  |  |  |  |  |  |  |  |  |  |  |  |  |  |
| LPS IgA(OD) |  |  |  | 0·07 |  |  | 0·07 |  |  |  | 0·70 |  |  | 0·62 |  |  |  | 0·38 |  |  | 0·40 |  |  |  | 0·39 |  |  | 0·51 |
| 1 (0·017-1·005) | 4/177 | Ref |  |  | Ref |  |  | 9/173 | Ref |  |  | Ref |  |  | 35/173 | Ref |  |  | Ref |  |  | 18/173 | Ref |  |  | Ref |  |  |
| 2 (1·010-1·465) | 8/176 | 2·01 | (0·62, 6·56) |  | 2·22 | (0·65, 7·62) |  | 13/168 | 1·49 | (0·65, 3·39) |  | 1·53 | (0·65, 3·59) |  | 27/168 | 0·79 | (0·50, 1·25) |  | 0·80 | (0·51, 1·27) |  | 31/168 | 1·77 | (1·03, 3·05) |  | 1·79 | (1·03, 3·11) |  |
| 3 (1·468-2·054) | 9/176 | 2·26 | (0·71, 7·22) |  | 2·29 | (0·69, 7·53) |  | 11/167 | 1·27 | (0·54, 2·98) |  | 1·22 | (0·53, 2·83) |  | 26/167 | 0·77 | (0·49, 1·22) |  | 0·77 | (0·49, 1·22) |  | 21/167 | 1·21 | (0·67, 2·19) |  | 1·13 | (0·62, 2·06) |  |
| 4 (2·054-3·441) | 11/176 | 2·77 | (0·90, 8·53) |  | 2·89 | (0·90, 9·27) |  | 8/165 | 0·93 | (0·37, 2·36) |  | 0·91 | (0·35, 2·37) |  | 39/165 | 1·17 | (0·78, 1·75) |  | 1·16 | (0·78, 1·73) |  | 26/165 | 1·51 | (0·86, 2·66) |  | 1·47 | (0·83, 2·62) |  |
|  |  |  |  |  |  |  |  |  |  |  |  |  |  |  |  |  |  |  |  |  |  |  |  |  |  |  |  |  |
| sCD14 (ng/mL) |  |  |  | 0·38 |  |  | 0·92 |  |  |  | 0·37 |  |  | 0·33 |  |  |  | 0·50 |  |  | 0·74 |  |  |  | 0·71 |  |  | 0·83 |
| 1 (16·770-2362·485) | 5/177 | Ref |  |  | Ref |  |  | 14/172 | Ref |  |  | Ref |  |  | 29/172 | Ref |  |  | Ref |  |  | 24/172 | Ref |  |  | Ref |  |  |
| 2 (2365·170-3270·630) | 7/176 | 1·41 | (0·46, 4·36) |  | 1·34 | (0·43, 4·19) |  | 10/169 | 0·73 | (0·33, 1·59) |  | 0·91 | (0·40, 2·06) |  | 33/169 | 1·16 | (0·74, 1·82) |  | 1·24 | (0·79, 1·96) |  | 20/169 | 0·85 | (0·49, 1·48) |  | 0·82 | (0·47, 1·42) |  |
| 3 (3271·670-5002·015) | 12/177 | 2·40 | (0·86, 6·68) |  | 1·89 | (0·66, 5·37) |  | 8/165 | 0·60 | (0·26, 1·38) |  | 0·59 | (0·23, 1·50) |  | 31/165 | 1·11 | (0·70, 1·76) |  | 1·25 | (0·78, 2·01) |  | 28/165 | 1·22 | (0·74, 2·01) |  | 1·11 | (0·67, 1·84) |  |
| 4 (5005·980-19607·785) | 8/176 | 1·61 | (0·54, 4·83) |  | 1·09 | (0·36, 3·30) |  | 9/168 | 0·66 | (0·29, 1·48) |  | 0·65 | (0·27, 1·59) |  | 34/168 | 1·20 | (0·77, 1·88) |  | 1·14 | (0·71, 1·83) |  | 24/168 | 1·02 | (0·61, 1·73) |  | 0·89 | (0·52, 1·54) |  |
|  |  |  |  |  |  |  |  |  |  |  |  |  |  |  |  |  |  |  |  |  |  |  |  |  |  |  |  |  |
| I-FABP (pg/mL) |  |  |  | **0·02** |  |  | **0·03** |  |  |  | 0·70 |  |  | 0·61 |  |  |  | 0·82 |  |  | 0·55 |  |  |  | 0·45 |  |  | 0·59 |
| 1 (1·385-737·365) | 5/177 | Ref |  |  | Ref |  |  | 10/172 | Ref |  |  | Ref |  |  | 33/172 | Ref |  |  | Ref |  |  | 24/172 | Ref |  |  | Ref |  |  |
| 2 (739·435-1346·800) | 5/176 | 1·01 | (0·30, 3·42) |  | 1·00 | (0·29, 3·39) |  | 8/171 | 0·80 | (0·33, 1·99) |  | 0·80 | (0·33, 1·95) |  | 33/171 | 1·01 | (0·65, 1·55) |  | 0·99 | (0·64, 1·54) |  | 21/171 | 0·88 | (0·51, 1·52) |  | 0·88 | (0·50, 1·55) |  |
| 3 (1349·415- 2280·515) | 9/177 | 1·80 | (0·61, 5·27) |  | 1·87 | (0·68, 5·12) |  | 13/168 | 1·33 | (0·60, 2·95) |  | 1·38 | (0·62, 3·07) |  | 31/168 | 0·96 | (0·62, 1·50) |  | 0·95 | (0·60, 1·49) |  | 25/168 | 1·07 | (0·63, 1·79) |  | 1·04 | (0·61, 1·76) |  |
| 4 (2280·515- 8338·955) | 13/176 | 2·61 | (0·95, 7·18) |  | 2·24 | (0·93, 5·42) |  | 10/163 | 1·06 | (0·45, 2·47) |  | 1·10 | (0·48, 2·52) |  | 30/163 | 0·96 | (0·61, 1·50) |  | 0·88 | (0·56, 1·38) |  | 26/163 | 1·14 | (0·68, 1·91) |  | 1·09 | (0·65, 1·82) |  |
|  |  |  |  |  |  |  |  |  |  |  |  |  |  |  |  |  |  |  |  |  |  |  |  |  |  |  |  |  |
| **Systemic inflammation** |  |  |  |  |  |  |  |  |  |  |  |  |  |  |  |  |  |  |  |  |  |  |  |  |  |  |  |  |
| AGP (g/L) |  |  |  | **0·03** |  |  | 0·24 |  |  |  | 0·86 |  |  | 0·96 |  |  |  | **0·02** |  |  | **0·04** |  |  |  | 0·80 |  |  | 0·50 |
| 1 (0·180-0·680) | 7/180 | Ref |  |  | Ref |  |  | 9/173 | Ref |  |  | Ref |  |  | 26/173 | Ref |  |  | Ref |  |  | 26/173 | Ref |  |  | Ref |  |  |
| 2 (0·685-0·920) | 4/173 | 0·59 | (0·18, 2·00) |  | 0·51 | (0·15, 1·77) |  | 12/169 | 1·36 | (0·59, 3·16) |  | 1·39 | (0·55, 3·53) |  | 31/169 | 1·22 | (0·76, 1·97) |  | 1·38 | (0·85, 2·26) |  | 23/169 | 0·91 | (0·54, 1·52) |  | 0·84 | (0·50, 1·42) |  |
| 3 (0·925-1·295) | 7/178 | 1·01 | (0·36, 2·83) |  | 0·70 | (0·25, 1·98) |  | 10/171 | 1·12 | (0·47, 2·70) |  | 1·12 | (0·39, 3·19) |  | 29/171 | 1·13 | (0·69, 1·83) |  | 1·22 | (0·73, 2·05) |  | 25/171 | 0·97 | (0·59, 1·62) |  | 0·86 | (0·48, 1·52) |  |
| 4 (1·300-8·040) | 14/175 | 2·06 | (0·85, 4·98) |  | 1·30 | (0·49, 3·43) |  | 10/161 | 1·19 | (0·50, 2·86) |  | 1·16 | (0·42, 3·23) |  | 41/161 | 1·69 | (1·09, 2·64) |  | 1·74 | (1·04, 2·90) |  | 22/161 | 0·91 | (0·54, 1·54) |  | 0·79 | (0·44, 1·41) |  |
|  |  |  |  |  |  |  |  |  |  |  |  |  |  |  |  |  |  |  |  |  |  |  |  |  |  |  |  |  |
| **Growth hormone axis** |  |  |  |  |  |  |  |  |  |  |  |  |  |  |  |  |  |  |  |  |  |  |  |  |  |  |  |  |
| IGF-1 (ng/mL) |  |  |  | 0·19 |  |  | 0·07 |  |  |  | 0·56 |  |  | 0·35 |  |  |  | 0·11 |  |  | 0·12 |  |  |  | 0·66 |  |  | 0·72 |
| 1 (0·375-95·535) | 10/177 | Ref |  |  | Ref |  |  | 12/167 | Ref |  |  | Ref |  |  | 40/167 | Ref |  |  | Ref |  |  | 23/167 | Ref |  |  | Ref |  |  |
| 2 (96·445-170·920) | 7/176 | 0·70 | (0·27, 1·81) |  | 0·63 | (0·25, 1·61) |  | 11/169 | 0·91 | (0·41, 2·00) |  | 0·88 | (0·39, 1·95) |  | 27/169 | 0·67 | (0·43, 1·03) |  | 0·64 | (0·41, 0·99) |  | 26/169 | 1·12 | (0·66, 1·88) |  | 1·13 | (0·67, 1·91) |  |
| 3 (171·065-253·650) | 11/177 | 1·10 | (0·48, 2·53) |  | 0·99 | (0·43, 2·29) |  | 8/166 | 0·67 | (0·28, 1·60) |  | 0·61 | (0·25, 1·46) |  | 34/166 | 0·86 | (0·57, 1·28) |  | 0·85 | (0·56, 1·28) |  | 18/166 | 0·79 | (0·44, 1·40) |  | 0·83 | (0·47, 1·46) |  |
| 4 (253·985-1144·363) | 4/176 | 0·40 | (0·13, 1·26) |  | 0·29 | (0·08, 1·02) |  | 10/172 | 0·81 | (0·36, 1·82) |  | 0·69 | (0·29, 1·66) |  | 26/172 | 0·63 | (0·40, 0·99) |  | 0·62 | (0·40, 0·98) |  | 29/172 | 1·22 | (0·74, 2·03) |  | 1·21 | (0·72, 2·01) |  |
|  |  |  |  |  |  |  |  |  |  |  |  |  |  |  |  |  |  |  |  |  |  |  |  |  |  |  |  |  |
| FGF21 (pg/mL) |  |  |  | 0·30 |  |  | 0·32 |  |  |  | 0·69 |  |  | 0·94 |  |  |  | 0·12 |  |  | 0·18 |  |  |  | **0·004** |  |  | **0·01** |
| 1 (0·465-245·185) | 5/177 | Ref |  |  | Ref |  |  | 9/172 | Ref |  |  | Ref |  |  | 28/172 | Ref |  |  | Ref |  |  | 19/172 | Ref |  |  | Ref |  |  |
| 2 (246·975-575·790) | 9/176 | 1·81 | (0·62, 5·30) |  | 2·14 | (0·80, 5·78) |  | 11/167 | 1·26 | (0·54, 2·96) |  | 1·20 | (0·52, 2·73) |  | 29/167 | 1·07 | (0·66, 1·71) |  | 1·07 | (0·66, 1·72) |  | 19/167 | 1·03 | (0·57, 1·88) |  | 1·04 | (0·57, 1·88) |  |
| 3 (576·350-1465·635) | 8/177 | 1·60 | (0·53, 4·80) |  | 1·56 | (0·54, 4·49) |  | 10/169 | 1·13 | (0·47, 2·71) |  | 1·02 | (0·40, 2·59) |  | 33/169 | 1·20 | (0·76, 1·89) |  | 1·16 | (0·72, 1·85) |  | 23/169 | 1·23 | (0·70, 2·18) |  | 1·18 | (0·66, 2·09) |  |
| 4 (1468·845-9228·637) | 10/176 | 2·01 | (0·70, 5·77) |  | 2·14 | (0·77, 5·90) |  | 11/166 | 1·27 | (0·54, 2·98) |  | 1·09 | (0·47, 2·54) |  | 37/166 | 1·37 | (0·88, 2·13) |  | 1·34 | (0·85, 2·10) |  | 35/166 | 1·91 | (1·14, 3·20) |  | 1·83 | (1·06, 3·15) |  |

‡Adjusted models adjusted for C-reactive protein, age, BMI, marital status, education, parity, SES, clinic site, WHO HIV stage, CD4 T-cell count, timing of ART initiation, infant sex, and regimen.

OD, optical density; IgA, immunoglobulin A; IgG, immunoglobulin G; LPS, lipopolysaccharide; sCD14, soluble CD14; I-FABP, intestinal fatty acid-binding protein; CRP, C-reactive protein; AGP, α1-acid glycoprotein; IGF-1, insulin-like growth factor 1; FGF21, fibroblast growth factor 21; ng, nanograms; pg, picograms; mg, milligrams; mL, milliliters; L, liters; EED, environmental enteric dysfunction

**Supplemental Table 9. Univariable and multivariable association of analyte concentrations (log_2_-transformed optical density or concentration) at 32 weeks gestation with infant birthweight, birthweight-for-gestational age z-score, and gestational age , among women living with HIV in Dar es Salaam Tanzania.^‡^ (I.e. one unit increase in log_2_ scale, or effect of doubling the biomarker concentration)**

|  | Birthweight | | | | | | Birthweight for gestational age z score | | | | | | Gestational age | | | | | |
| --- | --- | --- | --- | --- | --- | --- | --- | --- | --- | --- | --- | --- | --- | --- | --- | --- | --- | --- |
|  | Unadjusted | | | Adjusted | | | Unadjusted | | | Adjusted | | | Unadjusted | | | Adjusted | | |
|  | Mean diff (g) | 95% CI | p | Mean diff (g) | 95% CI | p | Mean diff (z-score) | 95% CI | p | Mean diff (z-score) | 95% CI | p | Mean diff (wks) | 95% CI | p | Mean diff (wks) | 95% CI | p |
| Flagellin IgG | 22 | (-44, 88) | 0·51 | 9 | (-59, 76) | 0·80 | 0·08 | (-0·11, 0·27) | 0·40 | 0·07 | (-0·13, 0·27) | 0·49 | -0·1 | (-0·4, 0·3) | 0·59 | -0·1 | (-0·5, 0·2) | 0·47 |
|  |  |  |  |  |  |  |  |  |  |  |  |  |  |  |  |  |  |  |
| Flagellin IgA | -22 | (-73, 29) | 0·40 | -20 | (-71, 30) | 0·43 | -0·02 | (-0·17, 0·13) | 0·79 | 0·00 | (-0·16, 0·15) | 0·95 | -0·2 | (-0·4, 0·1) | 0·20 | -0·2 | (-0·5, 0·1) | 0·18 |
|  |  |  |  |  |  |  |  |  |  |  |  |  |  |  |  |  |  |  |
| LPS IgG | 18 | (-45, 82) | 0·57 | 9 | (-55, 73) | 0·79 | 0·07 | (-0·12, 0·25) | 0·48 | 0·06 | (-0·13, 0·25) | 0·52 | -0·1 | (-0·4, 0·2) | 0·64 | -0·1 | (-0·4, 0·2) | 0·47 |
|  |  |  |  |  |  |  |  |  |  |  |  |  |  |  |  |  |  |  |
| LPS IgA | 1 | (-42, 43) | 0·97 | 4 | (-38, 47) | 0·85 | 0·02 | (-0·10, 0·15) | 0·71 | 0·04 | (-0·10, 0·17) | 0·59 | -0·1 | (-0·4, 0·1) | 0·20 | -0·1 | (-0·4, 0·1) | 0·22 |
|  |  |  |  |  |  |  |  |  |  |  |  |  |  |  |  |  |  |  |
| sCD14 | -22 | (-59, 15) | 0·24 | -19 | (-56, 18) | 0·31 | -0·04 | (-0·13, 0·05) | 0·40 | -0·04 | (-0·13, 0·05) | 0·40 | 0·0 | (-0·2, 0·1) | 0·75 | 0·0 | (-0·2, 0·2) | 0·74 |
|  |  |  |  |  |  |  |  |  |  |  |  |  |  |  |  |  |  |  |
| I-FABP | -2 | (-33, 29) | 0·89 | -0 | (-32, 32) | 0·99 | 0·00 | (-0·08, 0·09) | 0·91 | 0·01 | (-0·07, 0·09) | 0·76 | 0·0 | (-0·2, 0·1) | 0·57 | 0·0 | (-0·2, 0·1) | 0·58 |
|  |  |  |  |  |  |  |  |  |  |  |  |  |  |  |  |  |  |  |
| AGP | -69 | (-123,  -15) | **0·01** | -60 | (-113, -8) | **0·02** | -0·09 | (-0·23, 0·06) | 0·25 | -0·07 | (-0·22, 0·07) | 0·34 | -0·2 | (-0·5, 0·0) | 0·07 | -0·2 | (-0·5, 0·0) | 0·06 |
|  |  |  |  |  |  |  |  |  |  |  |  |  |  |  |  |  |  |  |
| CRP | -18 | (-42, 7) | 0·15 | -22 | (-45, 1) | 0·07 | -0·02 | (-0·09, 0·04) | 0·51 | -0·03 | (-0·09, 0·04) | 0·43 | -0·1 | (-0·2, 0·1) | 0·28 | -0·1 | (-0·2, 0·0) | 0·20 |
|  |  |  |  |  |  |  |  |  |  |  |  |  |  |  |  |  |  |  |
| IGF-1 | 18 | (-2, 38) | 0·08 | 17 | (-3, 37) | 0·09 | 0·05 | (0·00, 0·11) | **0·049** | 0·05 | (0·00, 0·11) | 0·06 | 0·0 | (-0·1, 0·1) | 0·50 | 0·0 | (-0·1, 0·1) | 0·59 |
|  |  |  |  |  |  |  |  |  |  |  |  |  |  |  |  |  |  |  |
| FGF21 | -26 | (-46, -5) | **0·01** | -26 | (-46, -6) | **0·01** | -0·02 | (-0·07, 0·04) | 0·54 | -0·02 | (-0·07, 0·04) | 0·56 | -0·1 | (-0·2, 0·0) | **0·008** | -0·1 | (-0·2, 0·0) | **0·009** |

‡Adjusted models adjusted for age, BMI, marital status, education, parity, SES, clinic site, WHO HIV stage, CD4 T-cell count, timing of ART initiation, infant sex, and regimen.

IgA, immunoglobulin A; IgG, immunoglobulin G; LPS, lipopolysaccharide; sCD14, soluble CD14; I-FABP, intestinal fatty acid-binding protein; CRP, C-reactive protein; AGP, α1-acid glycoprotein; IGF-1, insulin-like growth factor 1; FGF21, fibroblast growth factor 21

**Supplemental Table 10. Univariable and multivariable associations of analyte concentrations (log_2_-transformed optical density or concentration) at 32 weeks gestation with infant low birth weight (LBW), small-for-gestational age (SGA), and preterm birth, among 674 women living with HIV in Dar es Salaam Tanzania.^‡^ (I.e. one unit increase in log_2_ scale, or effect of doubling the biomarker concentration)**

|  | Stillbirth | | | | | | Low birth weight | | | | | | Small-for-gestational age | | | | | | Preterm birth | | | | | |
| --- | --- | --- | --- | --- | --- | --- | --- | --- | --- | --- | --- | --- | --- | --- | --- | --- | --- | --- | --- | --- | --- | --- | --- | --- |
|  | Unadjusted | | | Adjusted | | | Unadjusted | | | Adjusted | | | Unadjusted | | | Adjusted | | | Unadjusted | | | Adjusted | | |
|  | RR | 95% CI | p | RR | 95% CI | p | RR | 95% CI | p | RR | 95% CI | p | RR | 95% CI | p | RR | 95% CI | p | RR | 95% CI | p | RR | 95% CI | p |
| Flagellin IgG | 1·30 | (0·74, 2·28) | 0·36 | 1·24 | (0·68, 2·25) | 0·49 | 1·13 | (0·61, 2·10) | 0·70 | 1·11 | (0·54, 2·27) | 0·78 | 0·93 | (0·68, 1·27) | 0·64 | 0·92 | (0·66, 1·29) | 0·64 | 1·16 | (0·78, 1·74) | 0·46 | 1·14 | (0·78, 1·66) | 0·51 |
|  |  |  |  |  |  |  |  |  |  |  |  |  |  |  |  |  |  |  |  |  |  |  |  |  |
| Flagellin IgA | 1·66 | (0·96, 2·86) | 0·07 | 1·55 | (0·96, 2·51) | 0·07 | 1·22 | (0·80, 1·84) | 0·35 | 1·22 | (0·78, 1·92) | 0·39 | 0·98 | (0·77, 1·26) | 0·90 | 0·96 | (0·76, 1·23) | 0·76 | 1·09 | (0·83, 1·43) | 0·55 | 1·08 | (0·82, 1·41) | 0·60 |
|  |  |  |  |  |  |  |  |  |  |  |  |  |  |  |  |  |  |  |  |  |  |  |  |  |
| LPS IgG | 1·01 | (0·63, 1·62) | 0·95 | 0·88 | (0·57, 1·36) | 0·56 | 0·87 | (0·54, 1·40) | 0·57 | 0·88 | (0·53, 1·45) | 0·61 | 0·90 | (0·68, 1·19) | 0·47 | 0·91 | (0·67, 1·23) | 0·53 | 1·25 | (0·87, 1·79) | 0·23 | 1·24 | (0·86, 1·77) | 0·25 |
|  |  |  |  |  |  |  |  |  |  |  |  |  |  |  |  |  |  |  |  |  |  |  |  |  |
| LPS IgA | 1·58 | (1·04, 2·41) | **0·03** | 1·52 | (1·04, 2·23) | **0·03** | 0·99 | (0·73, 1·33) | 0·94 | 0·99 | (0·72, 1·36) | 0·95 | 0·92 | (0·76, 1·10) | 0·36 | 0·91 | (0·75, 1·10) | 0·34 | 1·06 | (0·84, 1·34) | 0·63 | 1·04 | (0·83, 1·29) | 0·76 |
|  |  |  |  |  |  |  |  |  |  |  |  |  |  |  |  |  |  |  |  |  |  |  |  |  |
| sCD14 | 1·29 | (0·88, 1·89) | 0·20 | 1·19 | (0·85, 1·66) | 0·31 | 1·01 | (0·78, 1·30) | 0·96 | 0·95 | (0·73, 1·25) | 0·73 | 1·11 | (0·94, 1·32) | 0·21 | 1·11 | (0·94, 1·31) | 0·21 | 0·97 | (0·80, 1·18) | 0·75 | 0·93 | (0·78, 1·12) | 0·45 |
|  |  |  |  |  |  |  |  |  |  |  |  |  |  |  |  |  |  |  |  |  |  |  |  |  |
| I-FABP | 1·40 | (1·03, 1·91) | **0·03** | 1·36 | (1·01, 1·85) | **0·04** | 1·08 | (0·84, 1·38) | 0·56 | 1·08 | (0·80, 1·44) | 0·62 | 0·97 | (0·86, 1·11) | 0·68 | 0·96 | (0·85, 1·10) | 0·58 | 1·07 | (0·92, 1·25) | 0·37 | 1·05 | (0·91, 1·21) | 0·53 |
|  |  |  |  |  |  |  |  |  |  |  |  |  |  |  |  |  |  |  |  |  |  |  |  |  |
| AGP | 1·45 | (0·95, 2·21) | 0·09 | 1·24 | (0·81, 1·90) | 0·32 | 1·06 | (0·74, 1·52) | 0·74 | 0·98 | (0·64, 1·49) | 0·92 | 1·22 | (0·99, 1·50) | 0·06 | 1·20 | (0·97, 1·48) | 0·09 | 1·07 | (0·83, 1·39) | 0·61 | 1·03 | (0·80, 1·34) | 0·80 |
|  |  |  |  |  |  |  |  |  |  |  |  |  |  |  |  |  |  |  |  |  |  |  |  |  |
| CRP | 1·21 | (0·93, 1·57) | 0·15 | 1·12 | (0·86, 1·46) | 0·41 | 1·01 | (0·83, 1·24) | 0·90 | 1·01 | (0·80, 1·26) | 0·96 | 1·08 | (0·96, 1·22) | 0·20 | 1·08 | (0·97, 1·21) | 0·16 | 1·02 | (0·90, 1·15) | 0·78 | 1·03 | (0·92, 1·17) | 0·59 |
|  |  |  |  |  |  |  |  |  |  |  |  |  |  |  |  |  |  |  |  |  |  |  |  |  |
| IGF-1 | 0·95 | (0·81,1·10) | 0·48 | 0·92 | (0·81, 1·06) | 0·25 | 1·08 | (0·92,1·27) | 0·34 | 1·06 | (0·88, 1·27) | 0·53 | 0·97 | (0·90,1·06) | 0·55 | 0·97 | (0·89, 1·06) | 0·54 | 1·05 | (0·91,1·21) | 0·52 | 1·04 | (0·90, 1·20) | 0·60 |
|  |  |  |  |  |  |  |  |  |  |  |  |  |  |  |  |  |  |  |  |  |  |  |  |  |
| FGF21 | 1·06 | (0·81,1·37) | 0·68 | 1·05 | (0·85, 1·30) | 0·63 | 1·10 | (0·95,1·29) | 0·20 | 1·09 | (0·91, 1·29) | 0·35 | 1·10 | (1·01,1·21) | **0·04** | 1·11 | (1·01, 1·22) | **0·03** | 1·14 | (1·02,1·27) | **0·03** | 1·12 | (1·00, 1·26) | **0·047** |

‡Adjusted models adjusted for age, BMI, marital status, education, parity, SES, clinic site, WHO HIV stage, CD4 T-cell count, timing of ART initiation, infant sex, and regimen.

IgA, immunoglobulin A; IgG, immunoglobulin G; LPS, lipopolysaccharide; sCD14, soluble CD14; I-FABP, intestinal fatty acid-binding protein; CRP, C-reactive protein; AGP, α1-acid glycoprotein; IGF-1, insulin-like growth factor 1; FGF21, fibroblast growth factor 21

**Supplemental Table 11. Univariable and multivariable associations of analyte concentrations (log_2_-transformed optical density or concentration) at 32 weeks gestation and associations with infant birthweight, birthweight-for-gestational age z-score, and gestational age, among women living with HIV in Dar es Salaam Tanzania, additionally adjusting for C-reactive protein.^‡^ (I.e. one unit increase in log_2_ scale, or effect of doubling the biomarker concentration)**

|  | Birthweight | | | | | | Birthweight for gestational age z score | | | | | | Gestational age | | | | | |
| --- | --- | --- | --- | --- | --- | --- | --- | --- | --- | --- | --- | --- | --- | --- | --- | --- | --- | --- |
|  | Unadjusted | | | Adjusted | | | Unadjusted | | | Adjusted | | | Unadjusted | | | Adjusted | | |
|  | Mean diff (g) | 95% CI | p | Mean diff (g) | 95% CI | p | Mean diff (z-score) | 95% CI | p | Mean diff (z-score) | 95% CI | p | Mean diff (wks) | 95% CI | p | Mean diff (wks) | 95% CI | p |
| Flagellin IgG | 22 | (-44, 88) | 0·51 | 15 | (-52, 81) | 0·67 | 0·08 | (-0·11, 0·27) | 0·40 | 0·08 | (-0·12, 0·28) | 0·44 | -0·1 | (-0·4, 0·3) | 0·59 | -0·1 | (-0·4, 0·2) | 0·56 |
|  |  |  |  |  |  |  |  |  |  |  |  |  |  |  |  |  |  |  |
| Flagellin IgA | -22 | (-73, 29) | 0·40 | -20 | (-69, 30) | 0·44 | -0·02 | (-0·17, 0·13) | 0·79 | 0·00 | (-0·16, 0·15) | 0·96 | -0·2 | (-0·4, 0·1) | 0·20 | -0·2 | (-0·5, 0·1) | 0·20 |
|  |  |  |  |  |  |  |  |  |  |  |  |  |  |  |  |  |  |  |
| LPS IgG | 18 | (-45, 82) | 0·57 | 11 | (-53, 75) | 0·74 | 0·07 | (-0·12, 0·25) | 0·48 | 0·06 | (-0·12, 0·25) | 0·50 | -0·1 | (-0·4, 0·2) | 0·64 | -0·1 | (-0·4, 0·2) | 0·50 |
|  |  |  |  |  |  |  |  |  |  |  |  |  |  |  |  |  |  |  |
| LPS IgA | 1 | (-42, 43) | 0·97 | 5 | (-37, 47) | 0·83 | 0·02 | (-0·10, 0·15) | 0·71 | 0·04 | (-0·09, 0·17) | 0·59 | -0·1 | (-0·4, 0·1) | 0·20 | -0·1 | (-0·4, 0·1) | 0·24 |
|  |  |  |  |  |  |  |  |  |  |  |  |  |  |  |  |  |  |  |
| sCD14 | -22 | (-59, 15) | 0·24 | -10 | (-49, 28) | 0·60 | -0·04 | (-0·13, 0·05) | 0·40 | -0·03 | (-0·13, 0·07) | 0·53 | 0·0 | (-0·2, 0·1) | 0·75 | 0·0 | (-0·2, 0·2) | 0·94 |
|  |  |  |  |  |  |  |  |  |  |  |  |  |  |  |  |  |  |  |
| I-FABP | -2 | (-33, 29) | 0·89 | 4 | (-29, 37) | 0·79 | 0·00 | (-0·08, 0·09) | 0·91 | 0·02 | (-0·06, 0·10) | 0·66 | 0·0 | (-0·2, 0·1) | 0·57 | 0·0 | (-0·2, 0·1) | 0·77 |
|  |  |  |  |  |  |  |  |  |  |  |  |  |  |  |  |  |  |  |
| AGP | -69 | (-123, -15) | **0·01** | -50 | (-111, 10) | 0·10 | -0·09 | (-0·23, 0·06) | 0·25 | -0·06 | (-0·23, 0·11) | 0·49 | -0·2 | (-0·5, 0·0) | 0·07 | -0·2 | (-0·5, 0·1) | 0·16 |
|  |  |  |  |  |  |  |  |  |  |  |  |  |  |  |  |  |  |  |
| IGF-1 | 18 | (-2, 38) | 0·08 | 18 | (-2, 38) | 0·08 | 0·05 | (0·00, 0·11) | **0·049** | 0·05 | (0·00, 0·11) | 0·06 | 0·0 | (-0·1, 0·1) | 0·50 | 0·0 | (-0·1, 0·1) | 0·63 |
|  |  |  |  |  |  |  |  |  |  |  |  |  |  |  |  |  |  |  |
| FGF21 | -26 | (-46,  -5) | **0·01** | -23 | (-43,  -3) | 0·02 | -0·02 | (-0·07, 0·04) | 0·54 | -0·01 | (-0·07, 0·04) | 0·65 | -0·1 | (-0·2,  0·0) | **0·008** | -0·1 | (-0·2, 0·0) | **0·02** |

‡Adjusted models adjusted for age, BMI, marital status, education, parity, SES, clinic site, WHO HIV stage, CD4 T-cell count, timing of ART initiation, infant sex, and regimen.

IgA, immunoglobulin A; IgG, immunoglobulin G; LPS, lipopolysaccharide; sCD14, soluble CD14; I-FABP, intestinal fatty acid-binding protein; CRP, C-reactive protein; AGP, α1-acid glycoprotein; IGF-1, insulin-like growth factor 1; FGF21, fibroblast growth factor 21

**Supplemental Table 12. Univariable and multivariable associations of analyte concentrations (log_2_-transformed optical density or concentration) at 32 weeks gestation and associations with infant low birth weight (LBW), small-for-gestational age (SGA), and preterm birth, among 674 women living with HIV in Dar es Salaam Tanzania, additionally adjusting for C-reactive protein.^‡^ (I.e. one unit increase in log_2_ scale, or effect of doubling the biomarker concentration)**

|  | Stillbirth | | | | | | Low birth weight | | | | | | Small-for-gestational age | | | | | | Preterm birth | | | | | |
| --- | --- | --- | --- | --- | --- | --- | --- | --- | --- | --- | --- | --- | --- | --- | --- | --- | --- | --- | --- | --- | --- | --- | --- | --- |
|  | Unadjusted | | | Adjusted | | | Unadjusted | | | Adjusted | | | Unadjusted | | | Adjusted | | | Unadjusted | | | Adjusted | | |
|  | RR | 95% CI | p | RR | 95% CI | p | RR | 95% CI | p | RR | 95% CI | p | RR | 95% CI | p | RR | 95% CI | p | RR | 95% CI | p | RR | 95% CI | p |
| Flagellin IgG | 1·30 | (0·74, 2·28) | 0·36 | 1·22 | (0·68, 2·18) | 0·50 | 1·13 | (0·61, 2·10) | 0·70 | 1·13 | (0·59, 2·16) | 0·70 | 0·93 | (0·68, 1·27) | 0·64 | 0·90 | (0·64, 1·26) | 0·52 | 1·16 | (0·78, 1·74) | 0·46 | 1·13 | (0·77, 1·64) | 0·53 |
|  |  |  |  |  |  |  |  |  |  |  |  |  |  |  |  |  |  |  |  |  |  |  |  |  |
| Flagellin IgA | 1·66 | (0·96, 2·86) | 0·07 | 1·56 | (0·97, 2·51) | 0·06 | 1·22 | (0·80, 1·84) | 0·35 | 1·22 | (0·80, 1·86) | 0·35 | 0·98 | (0·77, 1·26) | 0·90 | 0·96 | (0·76, 1·21) | 0·73 | 1·09 | (0·83, 1·43) | 0·55 | 1·07 | (0·82, 1·41) | 0·61 |
|  |  |  |  |  |  |  |  |  |  |  |  |  |  |  |  |  |  |  |  |  |  |  |  |  |
| LPS IgG | 1·01 | (0·63, 1·62) | 0·95 | 0·88 | (0·57, 1·35) | 0·55 | 0·87 | (0·54, 1·40) | 0·58 | 0·89 | (0·55, 1·43) | 0·63 | 0·90 | (0·68, 1·19) | 0·47 | 0·89 | (0·66, 1·21) | 0·47 | 1·25 | (0·87, 1·79) | 0·23 | 1·23 | (0·86, 1·76) | 0·26 |
|  |  |  |  |  |  |  |  |  |  |  |  |  |  |  |  |  |  |  |  |  |  |  |  |  |
| LPS IgA | 1·58 | (1·04, 2·41) | **0·03** | 1·53 | (1·05, 2·24) | **0·03** | 0·99 | (0·73, 1·33) | 0·94 | 0·99 | (0·73, 1·33) | 0·93 | 0·92 | (0·76, 1·10) | 0·36 | 0·91 | (0·75, 1·09) | 0·31 | 1·06 | (0·84, 1·34) | 0·63 | 1·03 | (0·83, 1·29) | 0·77 |
|  |  |  |  |  |  |  |  |  |  |  |  |  |  |  |  |  |  |  |  |  |  |  |  |  |
| sCD14 | 1·29 | (0·88, 1·89) | 0·20 | 1·14 | (0·79, 1·65) | 0.49 | 1·01 | (0·78, 1·30) | 0·96 | 0·95 | (0·73, 1·24) | 0·70 | 1·11 | (0·94, 1·32) | 0·21 | 1·07 | (0·91, 1·26) | 0·40 | 0·97 | (0·80, 1·18) | 0·75 | 0·91 | (0·76, 1·10) | 0·33 |
|  |  |  |  |  |  |  |  |  |  |  |  |  |  |  |  |  |  |  |  |  |  |  |  |  |
| I-FABP | 1·40 | (1·03, 1·91) | **0·03** | 1·34 | (0·99, 1·82) | 0·06 | 1·08 | (0·84, 1·38) | 0·56 | 1·09 | (0·84, 1·41) | 0·52 | 0·97 | (0·86, 1·11) | 0·68 | 0·95 | (0·83, 1·07) | 0·39 | 1·07 | (0·92, 1·25) | 0·37 | 1·04 | (0·90, 1·21) | 0·60 |
|  |  |  |  |  |  |  |  |  |  |  |  |  |  |  |  |  |  |  |  |  |  |  |  |  |
| AGP | 1·45 | (0·95, 2·21) | 0·09 | 1·17 | (0·73, 1·88) | 0·52 | 1·06 | (0·74, 1·52) | 0·74 | 0·97 | (0·61, 1·54) | 0·90 | 1·22 | (0·99, 1·50) | 0·06 | 1·14 | (0·90, 1·45) | 0·28 | 1·07 | (0·83, 1·39) | 0·61 | 1·00 | (0·74, 1·36) | 0·99 |
|  |  |  |  |  |  |  |  |  |  |  |  |  |  |  |  |  |  |  |  |  |  |  |  |  |
| IGF-1 | 0·95 | (0·81,1·10) | 0·48 | 0·91 | (0·80, 1·05) | 0·20 | 1·08 | (0·92,1·27) | 0·33 | 1·05 | (0·89, 1·23) | 0·55 | 0·97 | (0·90,1·06) | 0·55 | 0·97 | (0·88, 1·06) | 0·47 | 1·05 | (0·91,1·21) | 0·52 | 1·04 | (0·90, 1·20) | 0·61 |
|  |  |  |  |  |  |  |  |  |  |  |  |  |  |  |  |  |  |  |  |  |  |  |  |  |
| FGF21 | 1·06 | (0·81,1·37) | 0·68 | 1·04 | (0·84, 1·29) | 0·72 | 1·10 | (0·95,1·29) | 0·20 | 1·08 | (0·92, 1·26) | 0·35 | 1·10 | (1·01,1·21) | **0·04** | 1·10 | (1·00, 1·21) | 0·05 | 1·14 | (1·02,1·27) | **0·03** | 1·12 | (1·00, 1·26) | 0·05 |

‡Adjusted models adjusted for age, BMI, marital status, education, parity, SES, clinic site, WHO HIV stage, CD4 T-cell count, timing of ART initiation, infant sex, and regimen.

IgA, immunoglobulin A; IgG, immunoglobulin G; LPS, lipopolysaccharide; sCD14, soluble CD14; I-FABP, intestinal fatty acid-binding protein; CRP, C-reactive protein; AGP, α1-acid glycoprotein; IGF-1, insulin-like growth factor 1; FGF21, fibroblast growth factor 21

**Supplemental Table 13. Multivariable association of α1-acid glycoprotein (AGP) at 32 weeks gestation with infant birthweight, among 674 women living with HIV in Dar es Salaam Tanzania, comparing missing indicator method to multiple imputation method for maternal BMI and CD4 T-cell count.^‡^**

|  | Birthweight (g) | Missing indicator method | | | Multiple imputation method | | |
| --- | --- | --- | --- | --- | --- | --- | --- |
| AGP (g/L) | Mean (SD) | Mean diff (g) | 95% CI | p-value for trend | Mean diff (g) | 95% CI | p-value for trend |
| 1 (0·180-0·680) | 3233 (492) | Ref |  | 0·01 | Ref |  | 0·01 |
| 2 (0·685-0·920) | 3134 (474) | -122 | (-223, -21) |  | -129 | (-231, -27) |  |
| 3 (0·925-1·295) | 3160 (467) | -62 | (-161 ,37) |  | -74 | (-173, 24) |  |
| 4 (1·300-8·040) | 3065 (497) | -163 | (-269, -57) |  | -168 | (-274, -62) |  |

‡Adjusted models adjusted for age, BMI, marital status, education, parity, SES, clinic site, WHO HIV stage, CD4 T-cell count, timing of ART initiation, infant sex, and regimen.

The p-values were calculated from linear regression models.

**Supplemental Table 14. Multivariable association of insulin-like growth factor 1 (IGF-1) at 32 weeks gestation with infant birthweight, among 674 women living with HIV in Dar es Salaam Tanzania, comparing missing indicator method to multiple imputation method for maternal BMI and CD4 T-cell count.^‡^**

|  | Birthweight (g) | Missing indicator method | | | Multiple imputation method | | |
| --- | --- | --- | --- | --- | --- | --- | --- |
| IGF-1 (ng/mL) | Mean (SD) | Mean diff (g) | 95% CI | p-value for trend | Mean diff (g) | 95% CI | p-value for trend |
| 1 (0·375-95·535) | 3066 (503) | Ref |  | 0·003 | Ref |  | 0·002 |
| 2 (96·445-170·920) | 3140 (440) | 75 | (-23, 174) |  | 74 | (-26, 173) |  |
| 3 (171·065-253·650) | 3158 (462) | 93 | (-9, 195) |  | 92 | (-11, 195) |  |
| 4 (253·985-1144·363) | 3233 (520) | 172 | (64, 281) |  | 173 | (65, 282) |  |

‡Adjusted models adjusted for age, BMI, marital status, education, parity, SES, clinic site, WHO HIV stage, CD4 T-cell count, timing of ART initiation, infant sex, and regimen.

The p-values were calculated from linear regression models.

**Supplemental Table 15. Multivariable association of insulin-like growth factor 1 (IGF-1) at 32 weeks gestation with infant birthweight-for-gestational age (z-score), among 674 women living with HIV in Dar es Salaam Tanzania, comparing missing indicator method to multiple imputation method for maternal BMI and CD4 T-cell count.^‡^**

|  | Birthweight-for-gestational age (z-score) | Missing indicator method | | | Multiple imputation method | | |
| --- | --- | --- | --- | --- | --- | --- | --- |
| IGF-1 (ng/mL) | Mean (SD) | Mean diff (z-score) | 95% CI | p-value for trend | Mean diff (z-score) | 95% CI | p-value for trend |
| 1 (0·375-95·535) | -0·34 (1·31) | Ref |  | 0·01 | Ref |  | 0·01 |
| 2 (96·445-170·920) | -0·10 (1·17) | 0·25 | (-0·02, 0·51) |  | 0·24 | (-0·02, 0·51) |  |
| 3 (171·065-253·650) | -0·15 (1·27) | 0·19 | (-0·09, 0·47) |  | 0·19 | (-0·09, 0·47) |  |
| 4 (253·985-1144·363) | 0·08 (1·31) | 0·42 | (0·14, 0·70) |  | 0·42 | (0·15, 0·70) |  |

‡Adjusted models adjusted for age, BMI, marital status, education, parity, SES, clinic site, WHO HIV stage, CD4 T-cell count, timing of ART initiation, infant sex, and regimen.

The p-values were calculated from linear regression models.

**Supplemental Table 16. Multivariable association of fibroblast growth factor 21 (FGF21) at 32 weeks gestation with infant gestational age (weeks), among 674 women living with HIV in Dar es Salaam Tanzania, comparing missing indicator method to multiple imputation method for maternal BMI and CD4 T-cell count.^‡^**

|  | Gestational age (weeks) | Missing indicator method | | | Multiple imputation method | | |
| --- | --- | --- | --- | --- | --- | --- | --- |
| FGF21 (pg/mL) | Mean (SD) | Mean diff (g) | 95% CI | p-value for trend | Mean diff (g) | 95% CI | p-value for trend |
| 1 (0·465-245·185) | 39·6 (2·2) | Ref |  | 0·03 | Ref |  | 0·02 |
| 2 (246·975-575·790) | 39·4 (2·5) | -0·2 | (-0·7, 0·3) |  | -0·2 | (-0·7, 0·3) |  |
| 3 (576·350-1465·635) | 39·3 (2·2) | -0·3 | (-0·8, 0·2) |  | -0·3 | (-0·8, 0·1) |  |
| 4 (1468·845-9228·637) | 39·0 (2·6) | -0·6 | (-1·1, -0·1) |  | -0·7 | (-1·2, -0·1) |  |

‡Adjusted models adjusted for age, BMI, marital status, education, parity, SES, clinic site, WHO HIV stage, CD4 T-cell count, timing of ART initiation, infant sex, and regimen.

The p-values were calculated from linear regression models.

**Supplemental Table 17. Multivariable association of intestinal fatty acid-binding protein (I-FABP) at 32 weeks gestation with stillbirth, among 706 women living with HIV in Dar es Salaam Tanzania, comparing missing indicator method to multiple imputation method for maternal BMI and CD4 T-cell count.^‡^**

|  | Stillbirth | Missing indicator method | | | Multiple imputation method | | |
| --- | --- | --- | --- | --- | --- | --- | --- |
| I-FABP (pg/mL) | Events, n | RR | 95% CI | p-value for trend | RR | 95% CI | p-value for trend |
| 1 (1·385-737.365) | 5/177 | Ref |  | 0·02 | Ref |  | 0·01 |
| 2 (739.435-1346.800) | 5/176 | 1·06 | (0·32, 3·55) |  | 1·13 | (0·34, 3·77) |  |
| 3 (1349.415- 2280.515) | 9/177 | 2·04 | (0·75, 5·56) |  | 2·10 | (0·76, 5·78) |  |
| 4 (2280.515- 8338·955) | 13/176 | 2·44 | (1·00, 5·97) |  | 2·69 | (1·08, 6·73) |  |

‡Adjusted models adjusted for age, BMI, marital status, education, parity, SES, clinic site, WHO HIV stage, CD4 T-cell count, timing of ART initiation, infant sex, and regimen.

The p-values were calculated from log binomial models.

**Supplemental Table 18. Multivariable association of α1-acid glycoprotein (AGP) at 32 weeks gestation with small-for-gestational age (SGA), among 674 women living with HIV in Dar es Salaam Tanzania, comparing missing indicator method to multiple imputation method for maternal BMI and CD4 T-cell count.^‡^**

|  | Small-for-gestational age | Missing indicator method | | | Multiple imputation method | | |
| --- | --- | --- | --- | --- | --- | --- | --- |
| AGP (g/L) | Events, n | RR | 95% CI | p-value for trend | RR | 95% CI | p-value for trend |
| 1 (0·180-0·680) | 26/173 | Ref |  | 0·03 | Ref |  | 0·03 |
| 2 (0·685-0·920) | 31/169 | 1·31 | (0·81, 2·12) |  | 1·33 | (0·82, 2·14) |  |
| 3 (0·925-1·295) | 29/171 | 1·17 | (0·72, 1·90) |  | 1·18 | (0·73, 1·92) |  |
| 4 (1·300-8·040) | 41/161 | 1·69 | (1·07, 2·65) |  | 1·69 | (1·08, 2·65) |  |

‡Adjusted models adjusted for age, BMI, marital status, education, parity, SES, clinic site, WHO HIV stage, CD4 T-cell count, timing of ART initiation, infant sex, and regimen.

The p-values were calculated from log binomial models.

**Supplemental Table 19. Multivariable association of fibroblast growth factor 21 (FGF21) at 32 weeks gestation with preterm birth (<37 weeks), among 674 women living with HIV in Dar es Salaam Tanzania, comparing missing indicator method to multiple imputation method for maternal BMI and CD4 T-cell count.^‡^**

|  | Preterm birth (<37 weeks) | Missing indicator method | | | Multiple imputation method | | |
| --- | --- | --- | --- | --- | --- | --- | --- |
| FGF21 (pg/mL) | Events, n | RR | 95% CI | p-value for trend | RR | 95% CI | p-value for trend |
| 1 (0·465-245·185) | 19/172 | Ref |  | 0·02 | Ref |  | 0·01 |
| 2 (246·975-575·790) | 19/167 | 1·04 | (0·57, 1·90) |  | 1·06 | (0·58, 1·93) |  |
| 3 (576·350-1465·635) | 23/169 | 1·20 | (0·68, 2·11) |  | 1·22 | (0·69, 2·16) |  |
| 4 (1468·845-9228.637) | 35/166 | 1·77 | (1·04, 3·00) |  | 1·84 | (1·08, 3·13) |  |

‡Adjusted models adjusted for age, BMI, marital status, education, parity, SES, clinic site, WHO HIV stage, CD4 T-cell count, timing of ART initiation, infant sex, and regimen.

The p-values were calculated from log binomial models.

**Appendix 1 - The Micronutrient and EED Assessment Tool – Additional methods**

**Introduction**

The serum samples were tested on Quansys Biosciences’ (Logan, UT) Q-Plex^TM^ Human Environmental Enteric Dysfunction 11-plex ELISA array, also known as the Micronutrient and EED Assessment Tool (MEEDAT). Samples were received, counted, and stored at -80°C. The Q-Plex ^TM^ technology is traditional ELISA technology performed in micro scale and multiplexed allowing for the simultaneous measurement and detection of multiple proteins. It is an array of either sandwich ELISAs (IGF-1, FGF21, I-FABP, ferritin, sTfR, thyroglobulin, and HRP-2) or competitive ELISAs (AGP, CRP, sCD14, and RBP4). Both are quantitative as calibrator curves are used for data interpolation. For assays in the sandwich ELISA format, capture antibodies are printed in a 96-well microtitre plate and bind to target proteins in the sample matrix. After sample incubation, biotinylated secondary or detection antibodies bind to a different epitope on the target protein. Streptavidin-conjugated horseradish peroxidase then binds to the secondary antibodies and produces localized light when exposed to a chemiluminescent substrate. The signal produced is proportional to the amount of the protein in the sample matrix. Sample concentrations are calculated by fitting a 5-parameter logistic curve to the signal produced by the calibrator and back-calculating sample concentration based on measured signal. The competitive ELISA comprises a capture antibody printed into a 96-well microtitre plate which binds to the protein of interest. The sample is co-incubated with biotin-labeled protein that competes for binding sites on the printed capture antibody. This results in a measured signal that is inversely proportional to the concentration of the protein of interest in the samples [1].

**Materials and Methods**

***I. Technology Outline***

Q-Plex ^TM^ technology involves the micro-spotting of capture antibodies in discrete locations on the bottom of a 96 well plate, each spot being its own micro ELISA. Each well is identically spotted. Micro-spotted systems have the advantage of higher assay sensitivities and faster reaction kinetics due to minimizing diffusion constraints for analyte/antibody binding [2]. Standard ELISA incubation steps such as initial sample incubation, washing, secondary antibody incubation, washing, and incubation with the label and measurement are involved. The label and reporting system used in a Q-Plex^TM^ Array is chemiluminescent. Chemiluminescent ELISAs have been shown to be more sensitive than colorimetric detection systems [3-5].

***II. Assay Validation***

The Q-Plex ^TM^ kits used in the sample testing have undergone extensive validation. Ranges for each assay were determined by dilutions determining upper ranges where high end hook effect and apparent antibody saturation are avoided and lower ranges that are above detection limits [6]. Lower limits of detection (LLD) for each lot were calculated based off 2x the standard deviation of the background of 20 negative wells. Intra assay precision was measured with acceptance criteria of a coefficient of variation (CV) of less than 15%. Inter assay variability between plates was also determined to be less than 15% CV. Dilution linearity was measured in appropriate matrices and was between 80-120%. Measures of robustness and ruggedness includes testing with multiple technicians, multiple plates run by an individual technician, assay drift (wherein standard at high and low concentrations was measured to determine if the assay results could be affected by the addition of the aforementioned standard over a 20 min time frame) and edge effect (determining if a control gives a markedly different response based upon well location). All were determined to have a CV of less than 15%. As the technology is an array, all components were checked for cross reactivity with other components in the antigen and antibody cocktails and confirmed to have less than <0·5% cross-reactivity.

***III. Sample Preparation***

Samples were thawed at ambient temperature and then kept cold on ice. Thawed samples were then diluted with the appropriate Quansys sample dilution buffer. The sample buffers were formulated to reduce effects from heterophilic antibodies and other interferants [7]. Samples were diluted at a ratio (sample:total volume) of 1:10. Polypropylene low-binding 96-well plates were used to prepare the samples and standards prior to loading the Q-Plex TM plate. Each dilution was measured in duplicate for a total of 2 wells per sample. A multichannel pipettor was used in order to reduce pipetting error.

***IV. Image Analysis***

An image with a 270 second exposure time was captured using a Q-View^TM^ Imager LS and Q-View^TM^ Software. Levels of luminescent units or pixel intensity units were then measured by the Q-View^TM^ Software. The range of pixel intensity units is from 0 for black to 65535 (2^16^ for a 16 bit image).

***V. Standard Curve Fitting and Data Analysis***

The duplicate standard curves are fit by the Q-View^TM^ Software which allows for the selection of multiple non-linear and linear equations to fit the standard curve. Optimal curve fits are determined automatically by the software by evaluating recovery of the calibrator standards. The optimal dilution for samples are selected by the Q-View software, which finds the dilution where the pixel intensity values fall on the most linear portion of the standard curve.

**References**

1. Davies, C., Introduction to immunoassay principles, in The Immunoassay Handbook, Wild, D. Editor. 2005, Elsevier: Oxford, UK. p. 3-37.

2. Ekins, R., Ambient analyte assay. The Imunnoassay Handbook. Wild, D., Editor. 2005, Elsevier: Oxford, UK. p. 48-61.

3. Hatch, W., Scalarone, G., Comparison of colorimetric and chemiluminescent ELISAs for the detection of antibodies to Blastomyces dermatitidis. Journal of Medical and Biological Sciences, 2009. 3(1): p. 19-25.

4. Samineni, S., et al., Optimization, comparison, and application of colorimetric vs. chemiluminescence based indirect sandwich ELISA for measurement of human IL-23. J Immunoassay Immunochem, 2006. 27(2): p. 183-93.

5. Jantzie, L., Tanay, V., Todd, K., Methods in immunochemistry, in Handbook of neurochemistry and molecular neurobiology:Practical neurochemistry methods., A. Lajtha, Editor. 2007, Springer Science and Business Media, LLC: New York, NY. p. 193-218.

6. Kelley, M. and B. DeSilva, Key elements of bioanalytical method validation for macromolecules. Aaps J, 2007. 9(2): p. E156-63.

7. Bjerner, J., O.P. Bormer, and K. Nustad, *The war on heterophilic antibody interference.* Clin Chem, 2005. **51**(1): p. 9-11.
